# Structure–Activity Relationships of Hemocompatible Cationic 6-Azaindole Pyridinium Salts: Antimicrobial and Anticancer Activity

**DOI:** 10.3390/molecules31071220

**Published:** 2026-04-07

**Authors:** Roxana Ciorteanu, Ioana C. Marinas, Catalina Ionica Ciobanu, Ionel I. Mangalagiu, Ramona Danac

**Affiliations:** 1 ICI—RECENT AIR Center, Alexandru Ioan Cuza University of Iasi, 11 Carol I, 700506 Iasi, Romania; roxana.ciorteanu@uaic.ro; 2 Faculty of Chemistry, Alexandru Ioan Cuza University of Iasi, 11 Carol I, 700506 Iasi, Romania; 3 Research Institute of the University of Bucharest (ICUB), 91-95 Splaiul Independenței, 050095 Bucharest, Romania; ioana-cristina.marinas@icub.unibuc.ro; 4 Institute of Interdisciplinary Research—CERNESIM Centre, Alexandru Ioan Cuza University of Iasi, 11 Carol I, 700506 Iasi, Romania; catalina.ciobanu@uaic.ro

**Keywords:** 6-azaindole pyridinium salts, structure–activity relationships, antimicrobial, anticancer, ADME, hemocompatibility

## Abstract

A series of 6-azaindole pyridinium derivatives were synthesized, structurally characterized, and evaluated for their antimicrobial (against *Staphylococcus aureus*, *Escherichia coli*, and *Candida albicans*) and anticancer properties (against NCI 60 panel). Hemocompatibility was evaluated using the hemolytic index, while ADME properties were estimated using in silico methods. Structure–activity relationship analysis indicated that para-substitution of the phenyl ring, particularly with halogen or methoxy groups, influences antimicrobial activity, selectivity toward Gram-positive bacteria, and hemocompatibility. Compounds **2b** and **2c** showed the most notable antimicrobial effects, including inhibition of microbial adhesion at hemocompatible concentrations. Compound **2b** exhibited growth inhibition against cancer cells, showing 57% percent growth inhibition (PGI) against the MDA-MB-468 breast cancer cell line at 10 mM. Overall, these results highlight 6-azaindole pyridinium salts as a promising class of compounds for further investigation.

## 1. Introduction

The rapid emergence of antimicrobial resistance and the persistent global burden of cancer represent two of the most critical health challenges of the 21st century [1,2,3,4,5]. The increasing prevalence of multidrug-resistant bacterial strains, together with the limited introduction of novel antibiotic classes, has intensified the search for innovative antimicrobial chemotypes. Simultaneously, despite significant advances in oncology, drug resistance, systemic toxicity, and tumor heterogeneity continue to limit the long-term efficacy of many anticancer therapies [1,2,3,4,5,6,7]. These concerns underscore the urgent need for new molecular scaffolds with improved biological activity, optimized pharmacokinetic properties, and reduced adverse effects. In this context, the exploration of underinvestigated nitrogen-containing heterocycles represents a promising strategy for expanding both the antimicrobial and anticancer chemical space.

Nitrogen heterocycles are among the most prevalent structural motifs in approved pharmaceuticals and biologically active molecules [8,9,10,11]. The incorporation of nitrogen atoms into aromatic systems significantly modulates electronic distribution, hydrogen-bonding capacity, polarity, and solubility, often resulting in improved pharmacokinetic and pharmacodynamic profiles [8,9,10,11,12,13].

Azaindoles (pyrrolopyridines), which are bioisosteres of indole, represent an important class of fused N-heterocycles that have attracted sustained interest in medicinal chemistry in the recent years [14,15,16,17]. Among the four positional isomers (4-, 5-, 6-, and 7-azaindole), the 7-azaindole scaffold has been the most studied and incorporated into kinase inhibitors and antiviral agents [17,18,19]. In contrast, 4-, 5-, and particularly 6-azaindoles have been comparatively less explored, both from synthetic and biological perspectives.

However, the reports on the 6-azaindole scaffold have demonstrated its pharmacological relevance, as it has been incorporated into compounds exhibiting anticancer activity [15,20], clinically approved anti-HIV agents [14,21], selective DYRK1A inhibitors developed for type 1 diabetes [14,22], glucocorticoid receptor agonists with anti-inflammatory effects [14], URAT1 inhibitors for the treatment of hyperuricemia and gout [23], selective inhibitors targeting the hepatitis C virus NS4B protein [19], and glucocorticoid receptor agonists that showed in vitro activity and reduced collagen-induced arthritis in vivo [24]. Despite this demonstrated therapeutic versatility, the biological potential of 6-azaindole derivatives remains largely unexplored, with only limited and sporadic investigations reported to date. Nevertheless, 6-azaindole displays distinctive physicochemical properties, including favorable ionization behavior and the ability to form stable salts through protonation of the pyridine nitrogen, which may offer significant advantages in biological applications [16]. However, to the best of our knowledge, no biological studies have been reported for 6-azaindole-derived quaternary salts.

Given the urgent need for new antimicrobial and anticancer agents, and considering the privileged role of nitrogen-fused heterocycles in drug discovery, the development of novel 6-azaindole-based salts represents a promising yet insufficiently explored research direction. Building on our previous experience in the design and biological evaluation of pyridinium salts and other nitrogen-containing heterocycles with demonstrated antimicrobial and anticancer activity [25,26,27,28], we sought to extend this strategy to the 6-azaindole scaffold, aiming to further explore the therapeutic potential of heterocyclic salt systems.

## 2. Results and Discussion

### 2.1. Synthesis

**Scheme 1:**
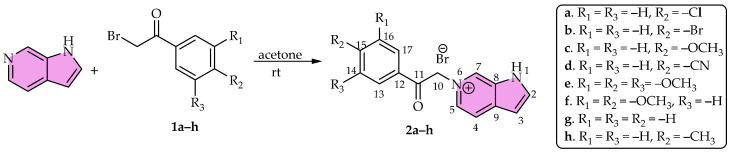
General scheme for the synthesis of monoquaternary salts **2a**–**h**.

The synthetic approach to synthesize 6-azaindole based pyridinium salts was the direct reaction of the corresponding substituted pyridine with various 2-bromoacetophenones **1** that differ by the substituent at the phenyl ring, the reactions being carried out in acetone, at room temperature (rt) (Scheme 1). The desired compounds precipitated directly from the reaction medium and were collected by filtration. The resulting solids were washed with acetone, and spectral analysis confirmed their high purity. The reactions afforded very good yields, ranging from 87% to 98%.

To the best of our knowledge, none of the eight synthesized monoquaternary salts have been reported in the literature. The structures of the synthesized compounds were proven by spectral (IR, ^1^H- and ^13^C-NMR) methods. The ^1^H- and ^13^C-NMR spectra of salts **2a**–**h** can be found in the Supplementary Materials (Figures S1–S16).

The ^1^H NMR spectra support the proposed 6-azaindole salt structure. Thus, the methylene singlet signal at δ = 6.46–6.58 ppm is consistent with its vicinal position to the positively charged cycloimmonium nitrogen and the carbonyl group, respectively, supporting successful salt formation. A more detailed analysis shows that the position of this signal is influenced by the electronic nature of the substituents on the aromatic ring. Electron-withdrawing groups such as cyano (4-CN) lead to a more pronounced deshielding effect, resulting in a downfield shift (δ = 6.58 ppm), while unsubstituted or weakly donating substituents (phenyl, p-tolyl) give intermediate values (δ = 6.54–6.55 ppm). In contrast, electron-donating substituents such as methoxy groups (4-OMe, 3,4-dimethoxy) tend to slightly shield the methylene protons, leading to upfield shifts (δ = 6.46 ppm). Halogen substituents (4-Cl, 4-Br) show only minor variations (δ = 6.46–6.50 ppm), reflecting the balance between their inductive electron-withdrawing and resonance-donating effects.

A downfield NH resonance is observed at 13.21–13.28 ppm, confirming the integrity of the azaindole core. The remaining azaindole and phenyl protons appear in the range of 9.36–7.00 ppm with the expected multiplicity pattern.

The ^13^C-NMR spectra further support the proposed structure, displaying the characteristic signals for carbonyl (190.2–192.0 ppm), aromatic (103.6–164.1 ppm) and methylene (64.8–65.4 ppm) carbon atoms. Overall, the spectroscopic data are in full agreement with the proposed 6-azaindole-derived salts structure.

### 2.2. In Silico Adsorption, Distribution, Metabolism, Excretion (ADME) Predictions

The predicted parameters of compounds **2a**–**h**, including molecular properties, pharmacokinetic profiles, drug-likeness, and medicinal chemistry characteristics, were evaluated, the results being summarized in Table 1.

The in silico ADME evaluation of compounds **2a**–**h** revealed favorable physicochemical and pharmacokinetic profiles across the entire series. The molecular weights (317.18–407.26 g/mol) and LogP values (0.82–2.43) fall within the acceptable range for orally active compounds, indicating balanced lipophilicity. All derivatives comply with Lipinski’s and Veber’s rules, with no violations observed, and present moderate water solubility (LogS between −5.35 and −4.38), supporting adequate oral bioavailability (predicted score: 0.55).

All compounds are predicted to exhibit high gastrointestinal absorption, while none are expected to permeate the blood–brain barrier, which may reduce the risk of central nervous system-related side effects. However, all derivatives are predicted to be P-gp substrates, which could influence their efflux and overall bioavailability. Importantly, no PAINS alerts were detected, suggesting a low probability of assay interference. A single Brenk alert was identified for all compounds, reflecting the permanently charged nature of quaternary ammonium moieties, which may limit membrane permeability and increase the likelihood of off-target interactions. However, the presence of the quaternary nitrogen does not preclude clinical utility, as several approved drugs contain this structural feature. The synthetic accessibility scores (1.85–2.54) indicate that the derivatives are relatively easy to synthesize.

Overall, the ADME profiles suggest that compounds **2a**–**h** possess suitable drug-like characteristics and favorable oral pharmacokinetic properties, supporting their further biological evaluation.

### 2.3. Biological Activity

#### 2.3.1. Antimicrobial Activity

This set of compounds, which share a similar 6-azaindole based pyridinium base, was tested for antimicrobial activity against *S. aureus*, *E. coli* and *C. albicans* reference strains.

The compounds’ overall polarity and the presence of halogenated substituents both had a significant impact on their antibacterial activity, according to the inhibition zone diameter (IZD) values. In the case of *S. aureus*, derivatives **2e** and **2h** generated the largest inhibition zones, suggesting an increased affinity for the Gram-positive strain and an efficient ability to diffuse in the solid medium. Compounds **2b** and **2h**, on the other hand, exhibited the highest activity against the *E. coli* strain, suggesting a more favorable interaction with the structural characteristics of Gram-negative bacteria, thereby highlighting the presence of some specific selectivity within the series. The compound **2h** exhibits an amphiphilic character, having both a polar component due to the cationic centre and a hydrophobic component conferred by the aromatic nucleus and the methyl group at position R_2_. Thus, both *S. aureus* and *E. coli* depend on the normal functioning of the membrane, which explains the activity against both types. Another hypothesis could be that the compound might inhibit fundamental processes common to both types of bacteria, such as: DNA replication (e.g., DNA gyrase), protein synthesis (ribosomes), central metabolism [29]. Gram-negative bacteria, such as *E. coli*, have an additional outer membrane that limits the access of many compounds; however, compound **2h** can cross this barrier (for example, through porins), exert its effect on them, or it may not be efficiently eliminated by bacterial efflux systems, allowing its intracellular accumulation [30,31]. Therefore, the activity of compound **2h** on both types of bacteria is likely explained by a combination of good cell permeability and a broad mechanism of action on common targets.

In general, the compounds showed moderate to low activity, with MIC values ranging between 0.625 and 2.5 mg/mL, demonstrating a bacteriostatic action that depends on the nature of the substituents at the base structure level.

Comparative study indicates that electronic and lipophilic changes to the aromatic ring have a significant impact on activity. Derivatives substituted with halogen groups (e.g., compound **2b**, Br) or methoxy (**2c**) demonstrated moderate antibacterial activity; however the addition of electron-donating substituents generally resulted in a decrease efficacy, as reflected by higher MIC values compared to halogenated derivatives [32,33]. Analysis of the data presented in Table 1 and Table 2 for the **2a**–**h** series highlights a correlation between chemical structure and antimicrobial activity. Compound **2b** bearing bromine as substituent on the phenyl ring exhibited the most favorable MIC value against *S. aureus* and *E. coli*, suggesting that this group promotes interactions with the microbial cell walls [34]. Derivatives with bulky groups or less electronegative substituents, such as compounds **2d** and **2e**, on the other hand, had lower activity, suggesting a lower affinity for the microbial cellular targets. Pyridinium cationic salts are a type of antibacterial substance that acts at the membrane level by electrostatically interacting with the negatively charged bacterial envelope, resulting in membrane instability and enhanced permeability [35,36].

In the case of *C. albicans*, only compounds **2a** and **2c** retain antifungal activity, suggesting that the presence of chlorine and methoxy fragments as substituents are advantageous for interactions with the fungal membrane [37]. Overall, more compact structures bearing electron-withdrawing substituents appear to provide the most favorable antimicrobial profile within the tested series, whereas modifications that reduce molecular polarity or flexibility led to diminished activity [38].

The MIC values revealed that the action was often stronger against *S. aureus* and *E. coli* than against *C. albicans*, indicating a potential selectivity against prokaryotic cells. The tested compounds were significantly less effective than gentamicin and ketoconazole. In comparison to the compounds in the green highlighted variants in Table 2, the DMSO control exhibited higher MIC values, indicating that the investigated chemical compounds were primarily responsible for the observed effects.

Most of the compounds exhibit relatively high MMC values against the investigated Gram-negative bacteria and yeasts, indicating a bacteriostatic effect rather than a bactericidal one. Compounds **2b** and **2c, however,** stand out in that their MMC values were closer to their corresponding MIC values, suggesting a higher microbicidal capacity than the others compounds.

The minimal biofilm eradication concentration (MBEC) results indicated that compounds **2b** and **2c** were also the most effective in inhibiting microbial adherence against *S. aureus*, while derivatives **2d** and **2h** showed their best MBEC values against *E coli*. (in the blue highlighted variants in Table 2). Compounds **2a**, **2e** and **2f** exhibit high MBEC values, indicating low efficiency in preventing microbial adherence. Furthermore, considerably reduced MBEC values compared to MIC for specific compounds (**2b**, **2c**, **2d**, **2h**) point to a putative mechanism of action involving biofilm matrix breakdown or cellular membrane contacts. The positive control for *C. albicans* did not exhibit the ability to adhere to an inert surface and therefore the influence of the compounds on the adhered biomass could not be quantified.

**Figure 1:**
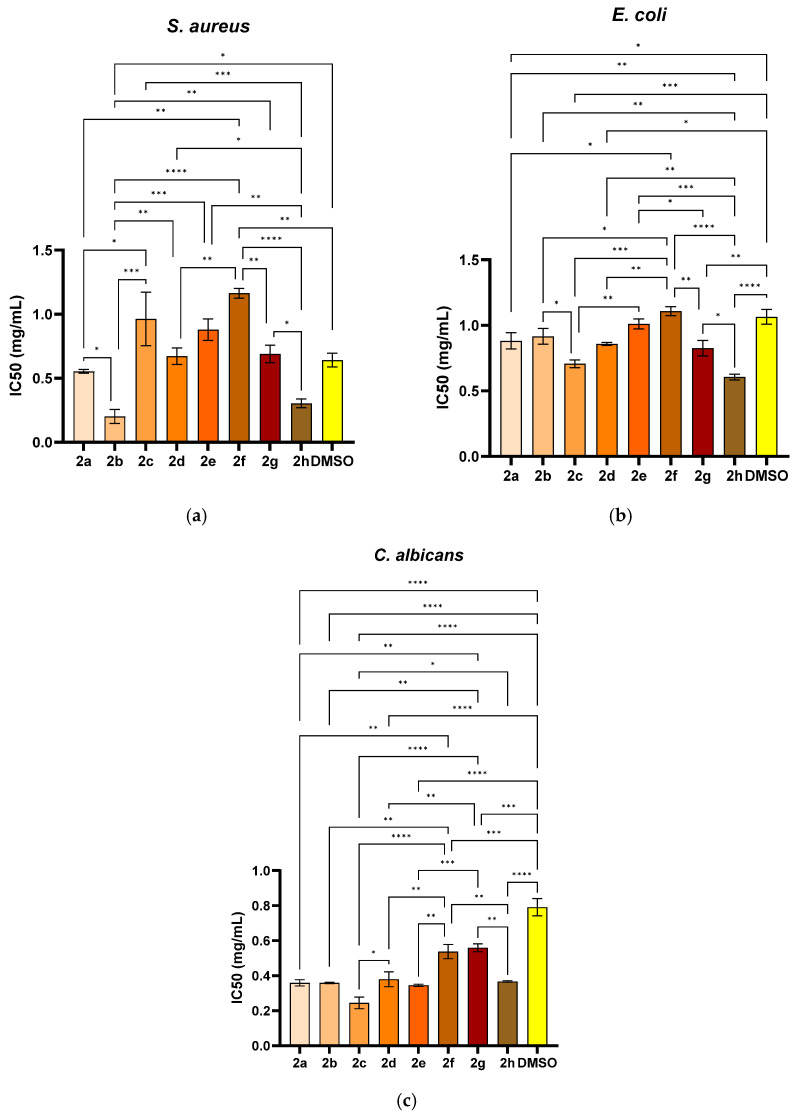
Antimicrobial activity expressed as IC_50_ values and correlated with the activity of the solvent for *S. aureus* (**a**), *E. coli* (**b**) and *C. albicans* (**c**). Data represent the mean of two independent experiments (*n* = 2), and error bars indicate standard deviation. Statistical significance was indicated as follows: * *p* < 0.05, ** *p* < 0.01, *** *p* < 0.001, **** *p* < 0.0001.

The graph in Figure 1a showed considerable differences in antibacterial activity between the series of derivatives against the Gram-positive pathogen *S. aureus*. With the lowest IC_50_ values, compounds **2b** and **2h** were found to exhibit an improved interaction profile with the cell wall of Gram-positive bacteria and efficient inhibition at low concentrations. On the other hand, compounds **2f** (*p* < 0.01) and **2c** (*p* > 0.05) exhibit less antibacterial activity and larger IC_50_ values in comparison to the solvent control. Within this group of compounds, the type of the substituents had a considerable impact on antibacterial activity, as seen by the numerous statistically significant comparisons.

The compounds differed significantly for the Gram-negative bacterium *E. coli* (Figure 1b), but not as much as for *S. aureus*. The lowest IC_50_ values were displayed by compounds **2h**, **2c**, and **2g**, indicating moderate efficacy against this microbial strain. In contrast, derivatives **2e** and **2f** had the highest IC_50_ values, which were comparable to those of DMSO (*p* > 0.05), resulting in reduced efficacy. The occurrence of several statistically significant changes suggested that structural modifications influence action against Gram-negative bacteria, but to a lesser extent than in *S. aureus*.

With the lowest IC_50_ values, **2a**, **2b**, **2c**, and **2e** showed the strongest antifungal activity within the series, indicating a more distinct antifungal profile (Figure 1c). Despite being much lower than DMSO’s IC_50_, compounds **2f** and **2g** displayed significantly greater IC_50_ values, suggesting decreased antifungal effectiveness (*p* < 0.001). The fact that the majority of changes were statistically significant emphasizes the susceptibility of antifungal activity to structural modifications in the substituent at the phenyl ring.

The compounds **2h**, **2b**, and **2c** were the most active within the tested series against all three tested microbial strains, according to the IC_50_ plots, while compounds **2f** and **2e** showed modest activity. The data collected suggests that local polarity could be one of the main factors contributing to the antibacterial effect. The lowest MIC, MBEC, and IC_50_ values were obtained for compounds with methoxy (**2c**), bromine (**2b**) and methyl (**2h**) substituents, indicating their crucial role in antibacterial efficacy.

**Figure 2:**
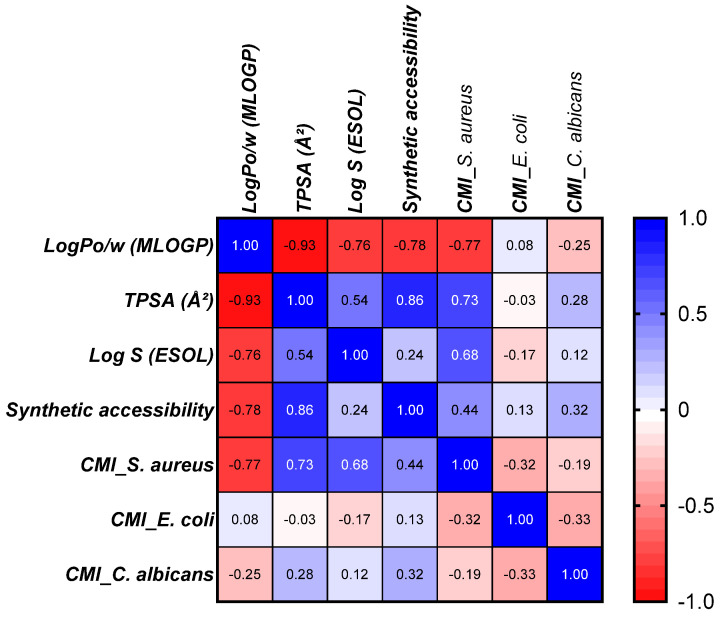
Pearson correlation heatmap between physicochemical descriptors and antimicrobial activity of the studied compounds.

Considering the structural properties of the compounds studied and the antimicrobial effects seen, a possible way they work is by interacting with the microbial cell membrane. Pyridinium salts, which have a positive charge, are known to interact with the negatively charged parts of the bacterial cell membrane [39,40,41]. This contact affects the membrane, increasing its permeability. The differential efficiency of these salts, based on their unique groups, suggests that their ability to interact with the membrane is regulated by their physicochemical properties, such as lipophilicity and polarity. However, these possibilities remain speculative in the absence of dedicated mechanistic studies. Lipophilicity (LogPo/w) and the MIC values against *S. aureus* showed a significant negative association (r = −0.77, *p* = 0.025) according to the Pearson correlation analysis; however, the topological polar surface area (TPSA) showed a positive correlation (r = 0.73, *p* = 0.039) (Figure 2).

This suggests that as lipophilicity increases, antimicrobial activity likely improves due to enhanced membrane permeability, a result confirmed by Podunavac-Kuzmanović et al. [42]. In the case of the antimicrobial activity of *E. coli* and *C. albicans* strains, no correlation was significant, indicating that these physicochemical parameters exert a reduced influence on these microorganisms. The Gram-positive and Gram-negative bacteria had distinct lipid compositions in their cell walls, which led to this interpretation. This conclusion was also mentioned by Biagi et al. [43] for cephalosporin and penicillin derivatives. Through this correlation, it can be concluded that hydrophobic molecules with a lower LogPo/w exhibit superior antibacterial properties, as they efficiently diffuse through Gram-positive bacteria cell walls. High TPSA values correlate positively with MIC value, suggesting that increased polarity diminishes antibacterial efficacy because more polar molecules tend to remain in aqueous environments, negatively affecting their permeability through bacterial membranes.

While significant correlations were present for *S. aureus*, they were insignificant for *E. coli* and *C. albicans*, suggesting that the permeability barriers of Gram-negative bacteria and yeasts are influenced by other additional structural factors. Thus, modifying the lipophilic-polar balance by adjusting the phenyl substituents can enhance antimicrobial efficacy, with compounds **2b** and **2c** being identified as the most active candidates for further biocompatibility research.

#### 2.3.2. Hemocompatibility

The hemolytic index and erythrocyte hemolysis measurements were used to evaluate the membrane cytotoxicity of the synthesized compounds. The findings revealed that all compounds showed low hemolytic activity, with hemolysis values of less than 5% at 2.5 and 5 mg/mL. At 2.5 mg/mL, dose-dependent hemolysis significantly decreased, while at 5 mg/mL, it increased. The experiment’s validity was demonstrated by the minimal hemolysis seen in the negative control (DMSO).

**Figure 3:**
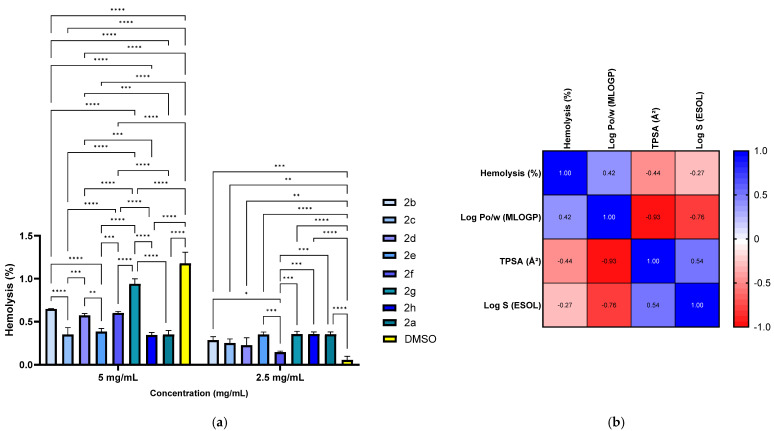
Hemolysis induced by compounds **2a**–**h** at two tested concentrations (**a**). Pearson correlation heatmap between physicochemical descriptors and hemolysis induced by analyses compounds (**b**). The results are in form: mean ± standard deviation, each experiment was performed in triplicate (* *p* < 0.05, ** *p* < 0.01, *** *p* < 0.001, **** *p* < 0.0001).

From the tested series, the highest hemolytic index, at a constant concentration of 5 mg/mL, was obtained for **2g** (0.94 ± 0.06%) followed by **2b** > **2f** > **2d** > **2e** > **2c** = **2a** > **2h** (0.35 ± 0.03%). Therefore, the unsubstituted phenyl by maximizing the hydrophobic character leads to a significant increase in the IH (*p* < 0.0001). By including halogenated substituents (Br, Cl), the lipophilicity increases [44], and the IH presented a Br > Cl trend (*p* < 0.0001). It was observed from Figure 3 a trend of increasing hemolysis for 3,4-dimethoxyphenyl substituted derivative **2f** compared to 3,4,5-trimethoxyphenyl substituted derivative **2e**, insignificant (*p* > 0.05), but significant compared to **2f** (*p* < 0.001). Between compounds **2a** and **2h**, the differences were insignificant at both concentrations, maintaining approximately constant values (*p* > 0.05). The homogeneity of variances was confirmed by the Spearman test for heteroscedasticity (*p* = 0.3253), and the distribution of residuals was considered acceptable for normality, validated by the Shapiro–Wilk test (*p* = 0.0776).

The Pearson correlation analysis, as depicted in Figure 3a, demonstrated a moderate positive correlation (r = 0.42) between lipophilicity, quantified by Log P, and the haemolytic index, which suggests that hydrophobic characteristics influence membrane interaction. Conversely, the negative correlation with TPSA (r = −0.44) implies that polarity mitigates haemolysis, thereby indicating that the observed effect was a result of an interaction among physicochemical parameters, rather than being exclusively determined by lipophilicity.

Previous studies on cationic surfactants based on pyridine [45,46,47], which usually have long aliphatic chains, have shown that haemolytic activity increases with lipophilicity due to enhanced interactions with erythrocyte membranes [46]. Even though the compounds studied in this research have different structures compared to those in this study and do not have long aliphatic chains, a similar process related to lipophilicity could be considered. Therefore, other factors, such as molecular polarity and the presence of aromatic groups, seem to be important.

The series of compounds **2a**–**h** inhibit penetration into lipid bilayers due to their ionic nature and reduced hemolytic indices [48]. Structural characteristics such as methoxy groups provided protection, although molecular weight had no clear relationship with hemolytic index. Overall, the data suggest that hemolytic activity was not solely determined by the lipophilic character of compounds. Instead, it was the product of a compromise between lipophilicity and molecular polarity. This is supported by a moderate correlation with Log P and an inverse correlation with TPSA. These factors together influence how a substance interacts with the red blood cell membrane.

#### 2.3.3. Anticancer Evaluation

Compounds **2a**–**e** were submitted and accepted for evaluation of their anticancer properties at a single high dose (10^−5^ M) against a panel of 60 human tumor cell lines at National Cancer Institute (NCI). These cell lines represent various cancer types, including leukemia, melanoma, and cancers of the lung, colon, central nervous system, ovary, kidney, prostate, and breast [49,50]. The anticancer screening results generated by the NCI are available in the Supplementary Material (Figures S17–S21).

Overall, the tested compounds demonstrated limited inhibitory effects at the screened concentration, with no compound reaching high or broad-spectrum growth inhibition. Among the evaluated series, compound **2b** exhibited the most pronounced inhibitory properties, producing 57% growth inhibition (PGI) against the MDA-MB-468 breast cancer cell line. In addition, compound **2b** showed moderate inhibition toward the HT-29 colon cancer cell line (PGI = 19%), while no significant inhibition was observed across the remaining panel. Compound **2a** demonstrated weaker activity, inhibiting MDA-MB-468 cells by 23% and LOX IMVI melanoma cells by 18%. Compounds **2c**, **2d** and **2e** were largely inactive across the panel, with their highest effect being modest inhibition (PGI ~ 18%) against CCRF-CEM leukemia cells for compounds **2c** and **2d**.

These findings indicate that, at 10^−5^ M, the evaluated compounds generally lack broad cytotoxic activity. However, the comparatively stronger inhibition of the MDA-MB-468 cell line by compound **2b** in the single-dose screening suggests a possible cell-line specific sensitivity. This observation may reflect differences in molecular targets, signaling pathways, or cellular uptake mechanisms in this triple-negative breast cancer model; however, further dose–response and mechanistic studies are required to confirm this behavior.

## 3. Materials and Methods

### 3.1. Chemistry

All commercially available reagents and solvents were used without further purification. Thin-layer chromatography (TLC) was performed on Merck 60F_254_ silica gel plates (Merck, Darmstadt, Germany). Visualization of the plates was achieved using a UV lamp (λ_max_ = 254 nm or 365 nm). Melting points were recorded on an A. Krüss Optronic Melting Point Meter KSP1N (Kruss, Hamburg, Germany) and are uncorrected. Proton and carbon nuclear magnetic resonance (NMR) spectra were recorded on a Bruker Avance III 500 MHz spectrometer (500 MHz, Bruker BioSpin GmbH, Rheinstetten, Germany), using DMSO-d6 as internal standards. Chemical shifts (δ) are reported in part per million (ppm) and coupling constants (*J*) in Hz. The following abbreviations are used to designate chemical shift multiplicities: s = singlet, d = doublet, t = triplet, q = quartet, m = multiplet, bs = broad singlet, dd = doublet of doublets. Infrared (IR) spectra were recorded as films with preparation performed by pelletizing with potassium bromide (KBr), on a Jasco 660 plus FTIR spectrophotometer (Jasco Corporation, Tokyo, Japan). Elemental analyses indicated by the symbols of the elements were within ± 0.4% of the theoretical values.

#### 3.1.1. General Procedure for Compounds 2

A solution of 6-azaindole (1 mmol, 1 equiv.) and the corresponding 2-bromoacetophenone (1.1 mmol, 1.1 equiv.) in approximately 10 mL of acetone was stirred at room temperature for 24 h. The resulting salt precipitated as a white to beige solid, was collected by filtration, and washed with acetone. No further purification was required, as the ^1^H and ^13^C-NMR spectra confirmed the formation and high purity of the obtained compounds.

#### 3.1.2. Physico-Chemical and Spectral Data of Compounds **2a**–**h**

6-(2-(4-chlorophenyl)-2-oxoethyl)-1H-pyrrolo[2,3-c]pyridin-6-ium bromide **2a**. White solid, η = 90%, m.p. = 219–220 °C; IR (KBr, ν(cm^−1^)): 3191, 3068, 2887, 2828, 1699, 1640, 1587, 1450, 1347, 1230, 1140, 1089, 982, 824. ^1^H-NMR (500 MHz, DMSO-d_6_), *δ*(ppm): 6.46 (s, 2H, H_10_), 7.02 (d, 1H, *J* = 2.5 Hz, H_3_), 7.76 (d, 2H, *J* = 8.5 Hz, H_14_, H_16_), 8.10 (d, 2H, *J* = 8.5 Hz, H_13_, H_17_), 8.22 (d, 1H, *J* = 6.5 Hz, H_4_), 8.32 (dd, 1H, *J* = 6.5; 1.0 Hz, H_5_), 8.42 (d, 1H, *J* = 3.0 Hz, H_2_), 9.27 (s, 1H, H_7_), 13.21 (s, 1H, H_1_). ^13^C-NMR (125 MHz, DMSO-d_6_), *δ*(ppm): 65.1 (C_10_), 103.7 (C_3_), 117.0 (C_4_), 129.3 (C_14_, C_16_), 130.1 (C_13_, C_17_), 131.2 (C_8_), 131.9 (C_7_), 132.6 (C_12_), 133.2 (C_5_), 136.1 (C_9_), 139.4 (C_15_), 140.3 (C_2_), 191.1 (C_11_). Anal. Calcd. for C_15_H_12_BrClN_2_O: C, 51.24; H, 3.44; N, 7.97. Found: C, 51.24; H, 3.42; N, 7.98.

6-(2-(4-bromophenyl)-2-oxoethyl)-1H-pyrrolo[2,3-c]pyridin-6-ium bromide **2b**. White solid, η = 94%, m.p. = 223–224 °C; IR (KBr, ν(cm^−1^)): 3190, 3050, 2886, 1697, 1639, 1582, 1449, 1348, 1230, 1139, 982, 822. ^1^H- NMR (500 MHz, DMSO-d_6_), *δ*(ppm): 6.50 (s, 2H, H_10_), 7.01 (d, 1H, *J* = 2.5 Hz, H_3_), 7.89 (d, 2H, *J* = 8.5 Hz, H_14_, H_16_), 8.02 (d, 2H, *J* = 8.5 Hz, H_13_, H_17_), 8.22 (d, 1H, *J* = 7.0 Hz, H_4_), 8.34 (dd, 1H, *J* = 7.0; 1.0 Hz, H_5_), 8.42 (d, 1H, *J* = 3.0 Hz, H_2_), 9.30 (s, 1H, H_7_), 13.24 (s, 1H, H_1_). ^13^C-NMR (125 MHz, DMSO-d_6_), *δ*(ppm): 65.0 (C_10_), 103.6 (C_3_), 116.9 (C_4_), 128.6 (C_15_), 130.2 (C_13_, C_17_), 131.2 (C_8_), 131.9 (C_7_), 132.2 (C_14_, C_16_), 132.9 (C_12_), 133.1 (C_5_), 136.1 (C_9_), 140.2 (C_2_), 191.3 (C_11_). Anal. Calcd. for C_15_H_12_Br_2_N_2_O: C, 45.49; H, 3.05; N, 7.07. Found: C, 45.48; H, 3.04; N, 7.08.

6-(2-(4-methoxyphenyl)-2-oxoethyl)-1H-pyrrolo[2,3-c]pyridin-6-ium bromide **2c**. White solid, η = 98%, m.p. = 233–234 °C; IR (KBr, ν(cm^−1^)): 3413, 3235, 3071, 2917, 2846, 1691, 1639, 1616, 1572, 1454, 1383, 1242, 1180, 1149, 616. ^1^H-NMR (500 MHz, DMSO-d_6_), *δ*(ppm): 3.90 (OMe), 6.46 (s, 2H, H_10_), 7.01 (d, 1H, *J* = 3.0 Hz, H_3_), 7.18 (d, 2H, *J* = 9.0 Hz, H_14_, H_16_), 8.06 (d, 2H, *J* = 9.0 Hz, H_13_, H_17_), 8.21 (d, 1H, *J* = 7.0 Hz, H_4_), 8.34 (dd, 1H, *J* = 6.5; 1.0 Hz, H_5_), 8.41 (d, 1H, *J* = 3.0 Hz, H_2_), 9.30 (s, 1H, H_7_), 13.22 (s, 1H, H_1_). ^13^C-NMR (125 MHz, DMSO-d_6_), *δ*(ppm): 55.8 (OMe), 64.8 (C_10_), 103.6 (C_3_), 114.4 (C_14_, C_16_), 116.9 (C_4_), 126.6 (C_12_), 130.7 (C_13_, C_17_), 131.2 (C_8_), 131.9 (C_7_), 133.2 (C_5_), 136.1 (C_9_), 140.1 (C_2_), 164.1 (C_15_), 190.2 (C_11_). Anal. Calcd. for C_16_H_15_BrN_2_O_2_: C, 55.35; H, 4.35; N, 8.07. Found: C, 55.36; H, 4.33; N, 8.10.

6-(2-(4-cyanophenyl)-2-oxoethyl)-1H-pyrrolo[2,3-c]pyridin-6-ium bromide **2d**. White solid, η = 98%, m.p. = 210–211 °C; IR (KBr, ν(cm^−1^)): 3194, 3087, 3055, 2881, 2227, 1706, 1640, 1450, 1349, 1226, 1142, 830. ^1^H-NMR (500 MHz, DMSO-d_6_), *δ*(ppm): 6.58 (s, 2H, H_10_), 7.01 (d, 1H, *J* = 3.0 Hz, H_3_), 8.15 (d, 2H, *J* = 8.5 Hz, H_14_, H_16_), 8.23–8.25 (overlapping signals, 3H, H_13_, H_17_, H_4_), 8.36 (d, 1H, *J* = 7.0 Hz, H_5_), 8.42 (d, 1H, *J* = 3.0 Hz, H_2_), 9.33 (s, 1H, H_7_), 13.27 (s, 1H, H_1_). ^13^C-NMR (125 MHz, DMSO-d_6_), *δ*(ppm): 65.4 (C_10_), 103.7 (C_3_), 116.2 (C_15_), 117.0 (C_4_), 118.1 (CN), 128.9 (C_13_, C_17_), 131.2 (C_8_), 131.9 (C_7_), 133.1 (C_14_, C_16_, C_5_), 136.2 (C_9_), 137.2 (C_12_), 140.3 (C_2_), 191.6 (C_11_). Anal. Calcd. for C_16_H_12_BrN_3_O: C, 56.16; H, 3.53; N, 12.28. Found: C, 56.16; H, 3.51; N, 12.29.

6-(2-oxo-2-(3,4,5-trimethoxyphenyl)ethyl)-1H-pyrrolo[2,3-c]pyridin-6-ium bromide **2e**. White solid, η = 97%, m.p. = 169–170 °C; IR (KBr, ν(cm^−1^)): 3104, 3065, 2946, 1694, 1647, 1586, 1463, 1352, 1316, 1148, 1128, 989, 838. ^1^H-NMR (500 MHz, DMSO-d_6_), *δ*(ppm): 3.79 (OMe), 3.91 (2 × OMe), 6.58 (s, 2H, H_10_), 7.02 (d, 1H, *J* = 3.0 Hz, H_3_), 7.40 (s, 2H, H_13_, H_17_), 8.23 (d, 1H, *J* = 7.0 Hz, H_4_), 8.35 (d, 1H, *J* = 7.0 Hz, H_5_), 8.41 (d, 1H, *J* = 3.0 Hz, H_2_), 9.32 (s, 1H, H_7_), 13.25 (s, 1H, H_1_). ^13^C-NMR (125 MHz, DMSO-d_6_), *δ*(ppm): 56.4 (2 × OMe), 60.4 (OMe), 65.2 (C_10_), 103.6 (C_3_), 106.0 (C_13_, C_17_), 117.0 (C_4_), 129.1 (C_12_), 131.3 (C_8_), 131.9 (C_7_), 133.1 (C_5_), 136.1 (C_9_), 140.2 (C_2_), 142.9 (C_15_), 153.0 (C_14_, C_16_), 191.0 (C_11_). Anal. Calcd. for C_18_H_19_BrN_2_O_4_: C, 53.09; H, 4.70; N, 6.88. Found: C, 53.08; H, 4.68; N, 6.90.

6-(2-(3,4-dimethoxyphenyl)-2-oxoethyl)-1H-pyrrolo[2,3-c]pyridin-6-ium bromide **2f**. White solid, η = 87%, m.p. = 160–161 °C; IR (KBr, ν(cm^−1^)): 3234, 3063, 2918, 2842, 1680, 1640, 1617, 1513, 1458, 1421, 1268, 1144, 1018. ^1^H-NMR (500 MHz, DMSO-d_6_), *δ*(ppm): 3.85 (OMe), 3.91 (OMe), 6.46 (s, 2H, H_10_), 7.01 (d, 1H, *J* = 3.0 Hz, H_3_), 7.22 (d, 1H, *J* = 8.5 Hz, H_16_), 7.53 (d, 1H, *J* = 2.0 Hz, H_13_), 7.79 (dd, 1H, *J* = 8.0; 2.0 Hz, H_17_), 8.21 (d, 1H, *J* = 7.0 Hz, H_4_), 8.32 (d, 1H, *J* = 6.5 Hz, H_5_), 8.41 (d, 1H, *J* = 3.0 Hz, H_2_), 9.28 (s, 1H, H_7_), 13.20 (s, 1H, H_1_). ^13^C-NMR (125 MHz, DMSO-d_6_), *δ*(ppm): 55.8 (OMe), 56.0 (OMe), 64.8 (C_10_), 103.6 (C_3_), 110.3 (C_13_), 111.3 (C_16_), 116.9 (C_4_), 123.3 (C_17_), 126.6 (C_12_), 131.2 (C_8_), 131.9 (C_7_), 133.2 (C_5_), 136.1 (C_9_), 140.2 (C_2_), 148.9 (C_14_), 154.2 (C_15_), 190.3 (C_11_). Anal. Calcd. for C_17_H_17_BrN_2_O_3_: C, 54.13; H, 4.54; N, 7.43. Found: C, 54.13; H, 4.52; N, 7.44.

6-(2-oxo-2-phenylethyl)-1H-pyrrolo[2,3-c]pyridin-6-ium bromide **2g**. White solid, η = 98%, m.p. 180–181 °C; IR (KBr, ν (cm^−1^)): 3413, 3235, 3065, 2939, 1705, 1684, 1640, 1617, 1455, 1383, 1231, 1147, 999, 827, 754. ^1^H-NMR (500 MHz, DMSO-d_6_), *δ*(ppm): 6.54 (s, 2H, H_10_), 7.01 (d, 1H, *J* = 2.5 Hz, H_3_), 7.66 (t, 2H, *J* = 7.5 Hz, H_14_, H_16_), 7.79 (t, 1H, *J* = 7.5 Hz, H_15_), 8.09 (d, 2H, *J* = 7.0 Hz, H_13_, H_17_), 8.22 (d, 1H, *J* = 6.5 Hz, H_4_), 8.37 (dd, 1H, *J* = 6.5; 0.5 Hz, H_5_), 8.42 (d, 1H, *J* = 3.0 Hz, H_2_), 9.33 (s, 1H, H_7_), 13.25 (s, 1H, H_1_). ^13^C-NMR (125 MHz, DMSO-d_6_), *δ*(ppm): 65.2 (C_10_), 103.6 (C_3_), 117.0 (C_4_), 128.3 (C_13_, C_17_), 129.1 (C_14_, C_16_), 131.2 (C_8_), 131.9 (C_7_), 133.2 (C_5_), 133.9 (C_12_), 134.6 (C_15_), 136.1 (C_9_), 140.2 (C_2_), 192.0 (C_11_). Anal. Calcd. for C_15_H_13_BrN_2_O: C, 56.80; H, 4.13; N, 8.83. Found: C, 56.79; H, 4.11; N, 8.85.

6-(2-oxo-2-(p-tolyl)ethyl)-1H-pyrrolo[2,3-c]pyridin-6-ium bromide **2h**. White solid, η = 98%, m.p. = 193–195 °C; IR (KBr, ν(cm^−1^)): 3412, 3205, 3036, 2925, 1691, 1641, 1604, 1457, 1349, 1230, 1140, 978, 810. ^1^H-NMR (500 MHz, DMSO-d_6_), *δ*(ppm): 2.42 (Me), 6.55 (s, 2H, H_10_), 7.00 (d, 1H, *J* = 2.5 Hz, H_3_), 7.44 (d, 2H, *J* = 8.0 Hz, H_14_, H_16_), 7.98 (d, 2H, *J* = 8.5 Hz, H_13_, H_17_), 8.21 (d, 1H, *J* = 6.5 Hz, H_4_), 8.38 (dd, 1H, *J* = 6.0; 0.5 Hz, H_5_), 8.41 (d, 1H, *J* = 2.5 Hz, H_2_), 9.36 (s, 1H, H_7_), 13.28 (s, 1H, H_1_). ^13^C-NMR (125 MHz, DMSO-d_6_), *δ*(ppm): 21.4 (Me), 65.0 (C_10_), 103.6 (C_3_), 116.9 (C_4_), 128.4 (C_13_, C_17_), 129.6 (C_14_, C_16_), 131.2 (C_8_), 131.4 (C_12_), 131.9 (C_7_), 133.2 (C_5_), 136.0 (C_9_), 140.1 (C_2_), 145.1 (C_15_), 191.5 (C_11_). Anal. Calcd. for C_16_H_15_BrN_2_O: C, 58.02; H, 4.57; N, 8.46. Found: C, 58.02; H, 4.54; N, 8.47.

### 3.2. In Silico ADME

The in silico ADME assessment of compounds **2a**–**h** was performed employing the SwissADME online platform (https://www.swissadme.ch/, accessed on 2 February 2026), focusing on molecular, pharmacokinetic, drug-likeness, and medicinal chemistry properties.

### 3.3. Biological Activity

#### 3.3.1. Antimicrobial Activity

##### Microbial Strains

For the evaluation of antimicrobial activity, the following reference strains were used: *Staphylococcus aureus* ATCC 25923, *Escherichia coli* ATCC 25922, and *Candida albicans* ATCC 10231. All reference strains were purchased from the American Type Culture Collection (ATCC, Washington, DC, USA).

##### Qualitative Evaluation of Antimicrobial Activity

Microbial suspensions were standardized to 1.5 × 10^8^ CFU/mL, equivalent to the 0.5 McFarland turbidity standard. These suspensions were derived from 18–24 h cultures cultured on solid media (Mueller-Hinton agar for bacteria and Sabouraud agar for yeast). In accordance with the criteria of the Clinical and Laboratory Standards Institute (CLSI), a diffusion-based approach was used to assess antimicrobial activity. Each compound’s stock solutions (10 mg/mL) were made in DMSO and a solvent control was used. Each stock solution was spotted over the agar surface that had been previously inoculated with the test strain in five microliters (5 μL). Following incubation, each spot’s diameter of the inhibition zone (DIZ) was measured in millimetres. Results were expressed as mean ± standard deviation (SD) for each substance tested in duplicate. Since the compounds were applied directly to the surface of the culture medium, the diameters of the inhibition zones were interpreted descriptively as follows: weak activity (<8 mm), moderate (8–15 mm), and high (>15 mm).

##### Quantitative Evaluation of Antimicrobial Activity

Quantitative analysis was performed using the serial two-fold microdilution method in liquid medium (Tryptone Soy Broth for bacteria and Sabouraud broth for yeasts) in 96-well microplates. Stock solutions (10 mg/mL) were prepared in DMSO, and the tested concentration range for each sample was 5000–0.078 µg/mL. A solvent control (DMSO) was included under identical experimental conditions. Each well was inoculated with 10 μL of microbial suspension adjusted to 1.5 × 10^8^ CFU/mL for bacteria and 1.5 × 10^6^ CFU/mL for yeasts, prepared from 18–24 h cultures. Microbial growth was assessed macroscopically and by measuring optical density at 620 nm after 24 h of incubation at 37 °C to determine the minimum inhibitory concentration (MIC), which is defined as the lowest concentration at which no visible microbial growth was observed or absorbance decreased to the sterility control level. GraphPad Prism 10.0 software’s Inhibitor vs. Response—Variable slope (four-parameter) function was used to evaluate the acquired data and determine IC_50_ values, which are the concentrations of samples that block 50% of microbial growth in comparison to the untreated inoculum.

##### Evaluation of Microbicidal Activity

The minimum microbicidal concentration (MMC) was ascertained by subculturing 5 µL samples from every well onto solid medium. For bacteria and yeasts, plates were incubated for 20–24 h at 37 °C. The MMC was defined as the lowest concentration at which no microbial growth was detected.

##### Microbial Adherence to Inert Surface

Microbial adherence to inert surface was assessed subsequent to quantitative antimicrobial analysis according to Marinas et al. [49]. In short, the method was carried out as follows: cold methanol fixation for 10 min, and 0.1% crystal violet staining for 15 min. The absorbance of the adhering biomass dyed and reconstituted in 33% acetic acid was measured at 490 nm.

#### 3.3.2. Hemocompatibility

The haemolytic index (HI) of the samples was measured using a spectrophotometric procedure, as described elsewhere [50]. A volume of nine mL of ram blood was collected in tubes with citrated dextrose (ACD) and centrifuged for 10 min at 8000 rpm, 4 °C. The supernatant was removed, and the sediment was washed three times with PBS (0.2 M, pH 7.4) before being resuspended in sterile saline solution (0.9%). In addition, 100 µL of the tested compound (5 mg/mL and 2.5 mg/mL in PBS starting from 10 mg/mL stock solution in DMSO, pH = 7.4) were added to 500 µL of erythrocyte suspension (10%). The mixtures were incubated for 1 h at 37 °C. The samples were centrifuged at 8000 rpm for 10 min, 4 °C, and the absorbance of the supernatant was measured at 540 nm. A solvent control (DMSO) was used for each concentration of the compound. Relative haemolysis was evaluated in comparison to haemolysis induced by 1% Triton X-100 (A+), which was set at 100%. For the negative control, PBS (A−) was used. Each set of experiments was conducted in triplicate, and the inhibitory activity was calculated as follows:

Haemolysis (%) = (A _sample_ − A_−_) × 100/(A_+_ − A_−_)

#### 3.3.3. Anticancer Activity

##### Cell Proliferation Assay

Compounds were tested against a panel of 60 human cancer cell lines at the National Cancer Institute, Rockville, MD. The anticancer activity was evaluated using the NCI-60 Human Tumor Cell Line Screen, a standardized panel of 60 well-characterized and authenticated human tumor cell lines maintained by the National Cancer Institute (NCI) Developmental Therapeutics Program (DTP). The cell lines used in this study belong to the National Cancer Institute (NCI) and are part of this screening platform (NCI-60 DTP Human Tumor Cell Line Screen database). The cell line information can be accessed through: NCI-60 DTP Human Tumor Cell Line Screen (https://dtp.cancer.gov/discovery_development/nci-60, accessed on 20 March 2026). Cytotoxicity experiments were performed using a 48 h exposure protocol consisting of a sulforhodamine B assay [51,52], described in detail in our previous work [27,53]. Growth inhibition was quantified using absorbance values from time zero (Tz), control growth (C), and treated cells (Ti). Percentage growth inhibition (PGI) was calculated as follows:

    PGI = [(Ti − Tz)/(C − Tz)] × 100, for Ti ≥ Tz

PGI = [(Ti − Tz)/Tz] × 100, for Ti < Tz

#### 3.3.4. Statistical Analysis

All data are expressed as mean ± standard deviation (SD). Antimicrobial experiments were performed in duplicate (each measurement was performed in triplicate as technical replicates), while hemocompatibility assays were conducted in triplicate. Statistical analysis was carried out using GraphPad Prism software (versions 9.0 and 10.0). For antimicrobial evaluation, IC_50_ values of the synthesized compounds were compared with the solvent control using one-way ANOVA followed by Tukey’s multiple comparison test with pooled variance. For hemocompatibility studies, two-way ANOVA with Geisser–Greenhouse correction followed by Tukey’s post hoc test was applied for multiple comparisons. Statistical significance was set at *p* < 0.05.

## 4. Conclusions

In this study, a series of 6-(2-oxo-2-(phenyl)ethyl)-1*H*-pyrrolo[2,3-c]pyridin-6-ium salts substituted with different functional groups on the phenyl ring, was synthesized in excellent yields (87−98%) using the direct reaction between 6-azaindole and various ω-bromoacetophenones. The 6-azaindole pyridinium salts **2a**–**h** were spectrally characterized and evaluated for the antimicrobial and anticancer properties, but also for their pharmacokinetic profile. Structure–activity relationship analysis suggested that the nature and electronic characteristics of the substituents significantly influence the observed biological activity. Compounds **2a**–**h** have demonstrated moderate to low antimicrobial activity, dependent on the nature of the substituents and the lipophilic-polar balance, with halogenated and methoxylated derivatives (especially **2b** and **2c**) standing out for their enhanced efficiency and hemocompatibility. The preliminary in vitro antitumor evaluation demonstrated that compound **2b** possesses selective moderate inhibitory properties against the MDA-MB-468 breast cancer cell line at 10^−5^ M (57% growth inhibition). The in silico ADME study indicated a generally favorable drug-likeness and predicted oral bioavailability of all synthesized compounds. Overall, our findings highlight the potential of 6-azaindole as promising bioactive scaffolds for the development of novel antimicrobial or anticancer agents.

## Figures and Tables

**Table 1:** In silico prediction of ADME parameters for compounds **2a**–**h**.

ADME Parameter	2a	2b	2c	2d
Physicochemical Properties				
Molecular weight	351.63 g/mol	396.08 g/mol	347.21 g/mol	342.19 g/mol
Log *P*_o/w_ (MLOGP)	2.31	2.43	1.46	1.12
Number of H-bond acceptors	1	1	2	2
Number of H-bond donors	1	1	1	1
Number of rotatable bonds	3	3	4	3
TPSA	36.74 Å^2^	36.74 Å^2^	45.97 Å^2^	60.53 Å^2^
Pharmacokinetics				
Gastrointestinal (GI) absorption	high	high	high	high
Blood–brain barrier (BBB) permeant	no	no	no	no
P-gp substrate	yes	yes	yes	yes
Drug likeness				
Log S (ESOL)	−5.04	−5.35	−4.50	−4.38
Water solubility class	moderately soluble	moderately soluble	moderately soluble	moderately soluble
Lipinski rule	no violation	no violation	no violation	no violation
Veber rule	no violation	no violation	no violation	no violation
Bioavailability	0.55	0.55	0.55	0.55
Medicinal Chemistry				
PAINS alerts	0	0	0	0
Brenk alerts	1: quaternary nitrogen	1: quaternary nitrogen	1: quaternary nitrogen	1: quaternary nitrogen
Synthetic accessibility	1.94	1.96	1.98	2.09
ADME parameter	**2e**	**2f**	**2g**	**2h**
Physicochemical Properties				
Molecular weight	407.26 g/mol	377.23 g/mol	317.18 g/mol	331.21 g/mol
Log *P*_o/w_ (MLOGP)	0.82	1.13	1.80	2.04
Number of H-bond acceptors	4	3	1	1
Number of H-bond donors	1	1	1	1
Number of rotatable bonds	6	5	3	3
TPSA	64.43 Å^2^	55.20 Å^2^	36.74 Å^2^	36.74 Å^2^
Pharmacokinetics				
Gastrointestinal (GI) absorption	high	high	high	high
Blood–brain barrier (BBB) permeant	no	no	no	no
P-gp substrate	yes	yes	yes	yes
Drug likeness				
Log S (ESOL)	−4.62	−4.56	−4.45	−4.75
Water solubility class	moderately soluble	moderately soluble	moderately soluble	moderately soluble
Lipinski rule	no violation	no violation	no violation	no violation
Veber rule	no violation	no violation	no violation	no violation
Bioavailability	0.55	0.55	0.55	0.55
Medicinal Chemistry				
PAINS alerts	0	0	0	0
Brenk alerts	1: quaternary nitrogen	1: quaternary nitrogen	1: quaternary nitrogen	1: quaternary nitrogen
Synthetic accessibility	2.54	2.32	1.85	1.96

**Table 2:** Quantitative and qualitative evaluation of antimicrobial activity expressed as inhibition zone diameter (mm), minimum inhibitory concentration (MIC), minimum microbicidal concentration (MMC) and the minimal biofilm eradication concentration (MBEC).

Sample	Analytical Parameter	*S. aureus*	*E. coli*	*C. albicans*	Sample	Analytical Parameter	*S. aureus*	*E. coli*	*C. albicans*
**2a**	MIC (mg/mL)	1.25	2.5	0.625	**2g**	MIC (mg/mL)	1.25	1.25	1.25
	MMC (mg/mL)	>2.5	2.5	2.5		MMC (mg/mL)	>2.5	>2.5	2.5
	MBEC (mg/mL)	2.5	2.5	nd		MBEC (mg/mL)	1.25	2.5	nd
	IZD (mm)	8.67 ± 0.58	7.33 ± 0.58	7.00 ± 0.00		IZD (mm)	6.00 ± 0.00	7.33 ± 0.58	6.00 ± 0.00
**2b**	MIC (mg/mL)	0.625	1.25	1.25	**2h**	MIC (mg/mL)	1.25	2.5	1.25
	MMC (mg/mL)	>2.5	1.25	1.25		MMC (mg/mL)	>2.5	2.5	2.5
	MBEC (mg/mL)	≤0.078	2.5	nd		MBEC (mg/mL)	1.25	≤0.078	nd
	IZD (mm)	8.00 ± 1.00	10.00 ± 0.0	8.67 ± 0.58		IZD (mm)	9.00 ± 1.00	7.67 ± 0.58	8.00 ± 0.00
**2c**	MIC (mg/mL)	2.5	1.25	0.625	Genta micin	MIC (mg/mL)	4.38	8.75	-
	MMC (mg/mL)	>2.5	2.5	1.25		MMC (mg/mL)	17.5	8.75	-
	MBEC (mg/mL)	≤0.078	2.5	nd		MBEC (mg/mL)	4.38	8.75	-
	IZD (mm)	6.67 ± 0.58	6.67 ± 0.58	5.67 ± 0.58		IZD (mm)	19.00 ± 1.41	19.50 ± 0.71	-
**2d**	MIC (mg/mL)	2.5	1.25	1.25	Ketoconazole	MIC (mg/mL)	-	-	17.5
	MMC (mg/mL)	>2.5	2.5	2.5		MMC (mg/mL)	-	-	7
	MBEC (mg/mL)	2.5	≤0.078	nd		MBEC (mg/mL)	-	-	nd
	IZD (mm)	6.33 ± 1.16	6.00 ± 0.00	6.67 ± 0.58		IZD (mm)	-	-	8.00 ± 0.00
**2e**	MIC (mg/mL)	2.5	1.25	1.25	DMSO	MIC (mg/mL)	2.5	2.5	1.25
	MMC (mg/mL)	>2.5	2.5	1.25		MMC (mg/mL)	>2.5	>2.5	>2.5
	MBEC (mg/mL)	2.5	2.5	nd		MBEC (mg/mL)	1.25	1.25	nd
	IZD (mm)	9.33 ± 1.16	6.00 ± 0.00	7.33 ± 0.58		IZD (mm)	6.00 ± 0.00	6.00 ± 0.00	7.67 ± 0.58
**2f**	MIC (mg/mL)	1.25	1.25	1.25					
	MMC (mg/mL)	>2.5	2.5	1.25					
	MBEC (mg/mL)	2.5	2.5	nd					
	IZD (mm)	5.00 ± 0.00	6.33 ± 0.58	7.67 ± 1.53					

nd—not determined.

## Data Availability

The data presented in this study are available on request from the corresponding author.

## Supplementary Materials

The following supporting information can be downloaded at: https://www.mdpi.com/article/10.3390/molecules31071220/s1; Figures S1–S16: ^1^H and ^13^C NMR-spectra of compounds **2a**–**h**; Figures S17–S21: The anticancer screening results generated by the NCI for compounds **2a**–**e**.
