# Supplementary material for: Structure–Activity Relationships of Hemocompatible Cationic 6-Azaindole Pyridinium Salts: Antimicrobial and Anticancer Activity
Source: Molecules. 2026 Apr 7;31(7):1220. doi: 10.3390/molecules31071220 (PMC13074213; doi:10.3390/molecules31071220)
Supplement: Supplementary file 1 [file molecules-31-01220-s001.zip › molecules-4212574-supplementary.pdf]

Supplementary material

for

Structure–Activity Relationships of Hemocompatible Cationic 6-Azaindole Pyridinium Salts: Antimicrobial and Anticancer Activity

Roxana Ciorteanu[1,2], Ioana C. Marinas[3], Catalina Ionica Ciobanu[4], Ionel I. Mangalagiu[2,*], Ramona Danac[2,*]

[1]Alexandru Ioan Cuza University of Iasi, ICI - RECENT AIR Center, 11 Carol I, Iasi 700506, Romania; roxana.ciorteanu@uaic.ro (R.C)

[2]Faculty of Chemistry, Alexandru Ioan Cuza University of Iasi, 11 Carol I, Iasi 700506, Romania; roxana.ciorteanu@uaic.ro (R.C); ionelm@uaic.ro (I.I.M.); rdanac@uaic.ro (R.D.)

[3]Research Institute of the University of Bucharest-ICUB, 90-92 Sos. Panduri, 5th District, Bucharest 050095, Romania; ioana-cristina.marinas@icub.unibuc.ro (I.C.M.);

[4]Institute of Interdisciplinary Research - CERNESIM Centre, Alexandru Ioan Cuza University of Iasi, 11 Carol I, Iasi 700506, Romania; catalina.ciobanu@uaic.ro (C.I.C)*

Correspondence: rdanac@uaic.ro (R.D.); ionelm@uaic.ro (I.I.M.)

Table of Contents

Figure S1. ^{1}H-NMR (500 MHz, DMSO-d$_6$) spectrum of compound **2a** ………………………….3

Figure S2. ^{13}C-NMR (125 MHz, DMSO-d$_6$) spectrum of compound **2a** ……………………......3

Figure S3. ^{1}H-NMR (500 MHz, DMSO-d$_6$) spectrum of compound **2b** …………,…………....4

Figure S4. ^{13}C-NMR (125 MHz, DMSO-d$_6$) spectrum of compound **2b** ……………….....…..4

Figure S5. ^{1}H-NMR (500 MHz, DMSO-d$_6$) spectrum of compound **2c** …………………...…...5

Figure S6. ^{13}C-NMR (125 MHz, DMSO-d$_6$) spectrum of compound **2c** ……………….....…..5

Figure S7. ^{1}H-NMR (500 MHz, DMSO-d$_6$) spectrum of compound **2d** ……………..……..…….6

Figure S8. ^{13}C-NMR (125 MHz, DMSO-d$_6$) spectrum of compound **2d** ……………………….....6

Figure S9. ^{1}H-NMR (500 MHz, DMSO-d$_6$) spectrum of compound **2e** ……...……...…..……7

Figure S10. ^{13}C-NMR (125 MHz, DMSO-d$_6$) spectrum of compound **2e** …………….....…...7

Figure S11. ^{1}H-NMR (500 MHz, DMSO-d$_6$) spectrum of compound **2f** ……………......…..8

Figure S12. ^{13}C-NMR (125 MHz, DMSO-d$_6$) spectrum of compound **2f** …………….....…...8

Figure S13. ^{1}H-NMR (500 MHz, DMSO-d$_6$) spectrum of compound **2g** …………...………..9

Figure S14. ^{13}C-NMR (125 MHz, DMSO-d$_6$) spectrum of compound **2g** ……………….....….9

Figure S15. ^{1}H-NMR (500 MHz, DMSO-d$_6$) spectrum of compound **2h** ………..………...10

Figure S16. ^{13}C-NMR (125 MHz, DMSO-d$_6$) spectrum of compound **2h** …………….....…10

Figure S17. Results of the *in vitro* growth of cancer cell lines in the single-dose assay for compound **2a**…………………………………………………………………………...**11**

Figure S18. Results of the *in vitro* growth of cancer cell lines in the single-dose assay for compound **2b**……………………………………………………………………………..………12

Figure S19. Results of the *in vitro* growth of cancer cell lines in the single-dose assay for compound **2c**………………………………………………………………………………………13

Figure S20. Results of the *in vitro* growth of cancer cell lines in the single-dose assay for compound **2d**……………………………………………………………………………..……14

Figure S21. Results of the *in vitro* growth of cancer cell lines in the single-dose assay for compound **2e**………………………………………………………………………………………15

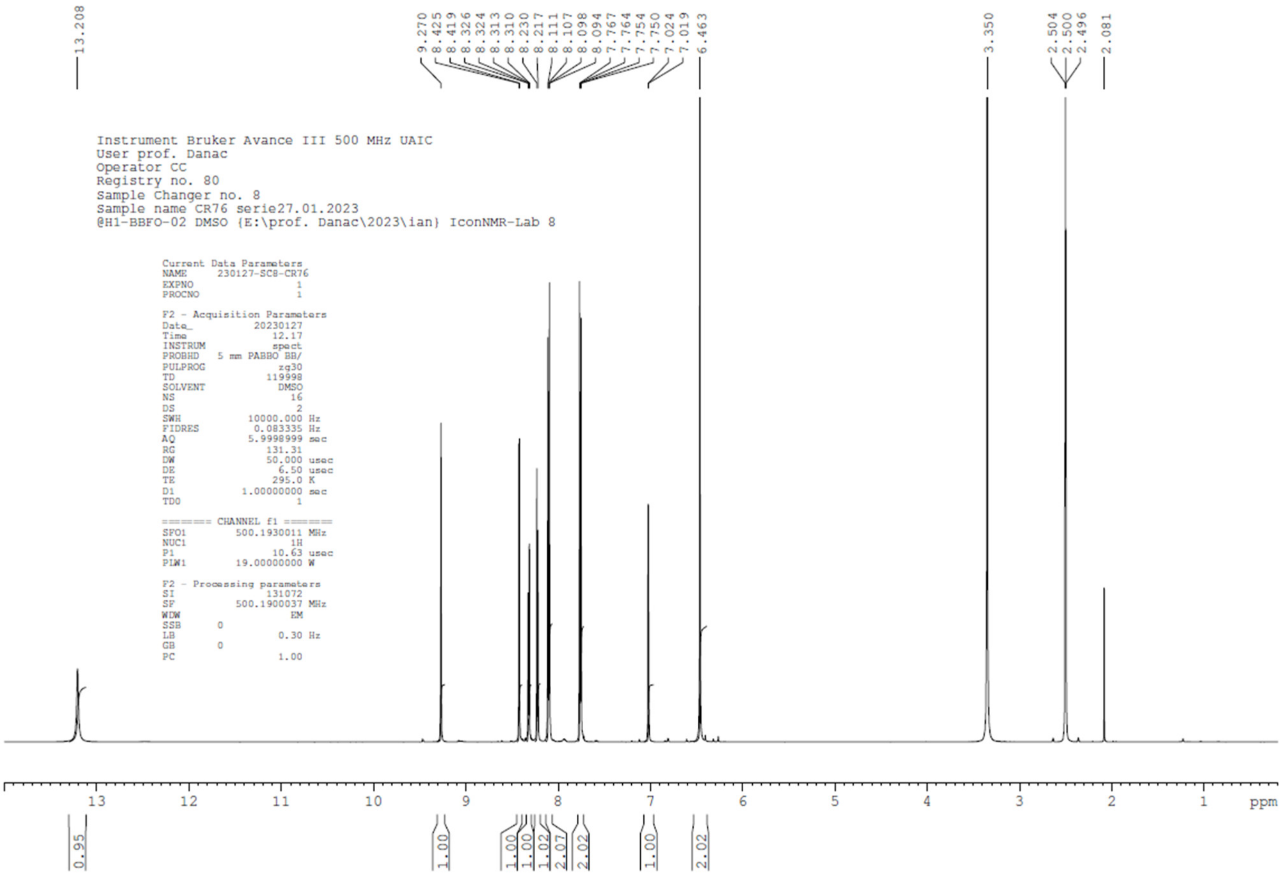

Figure S1. ^{1}H-NMR (500 MHz, DMSO-d$_6$) spectrum of compound **2a**

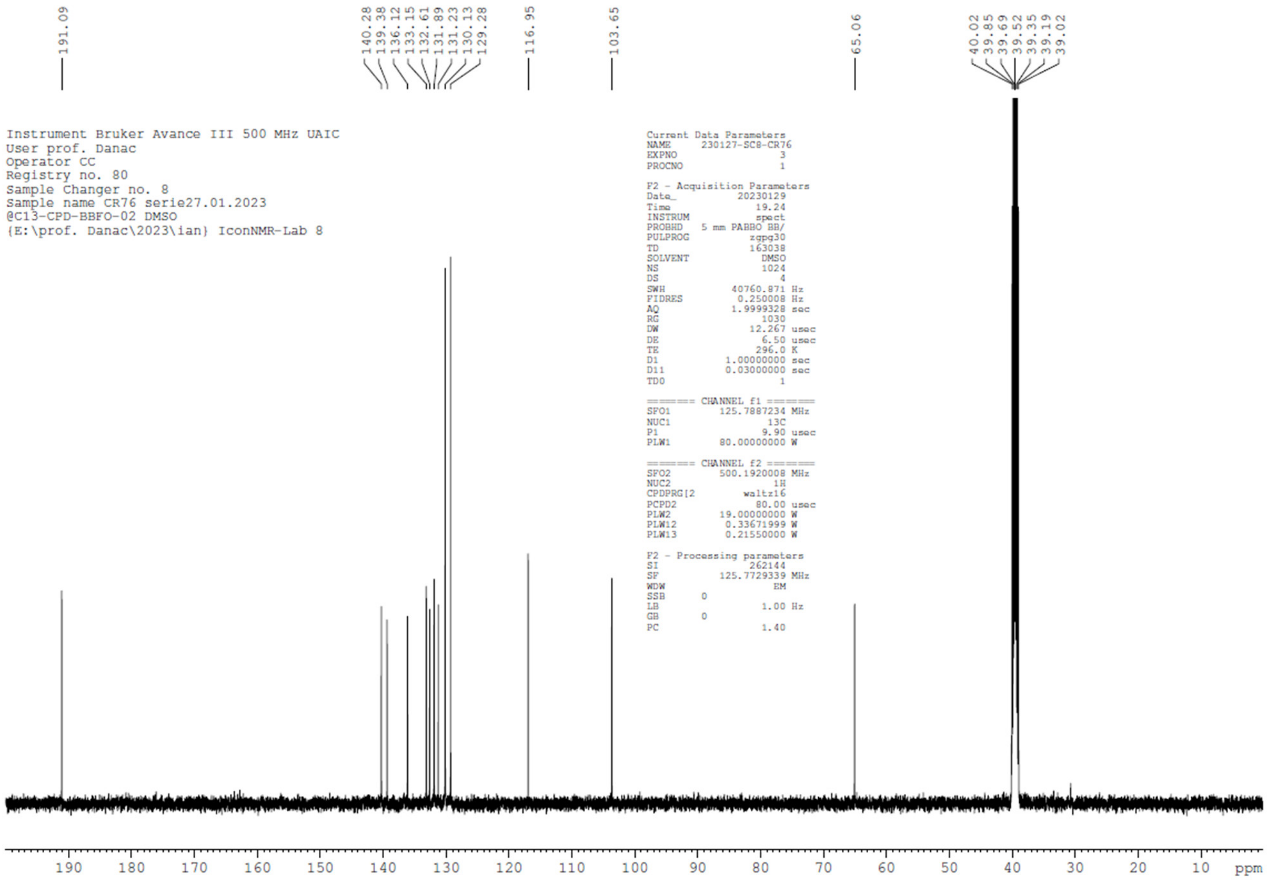

Figure S2. ^{13}C-NMR (125 MHz, DMSO-d$_6$) spectrum of compound **2a**

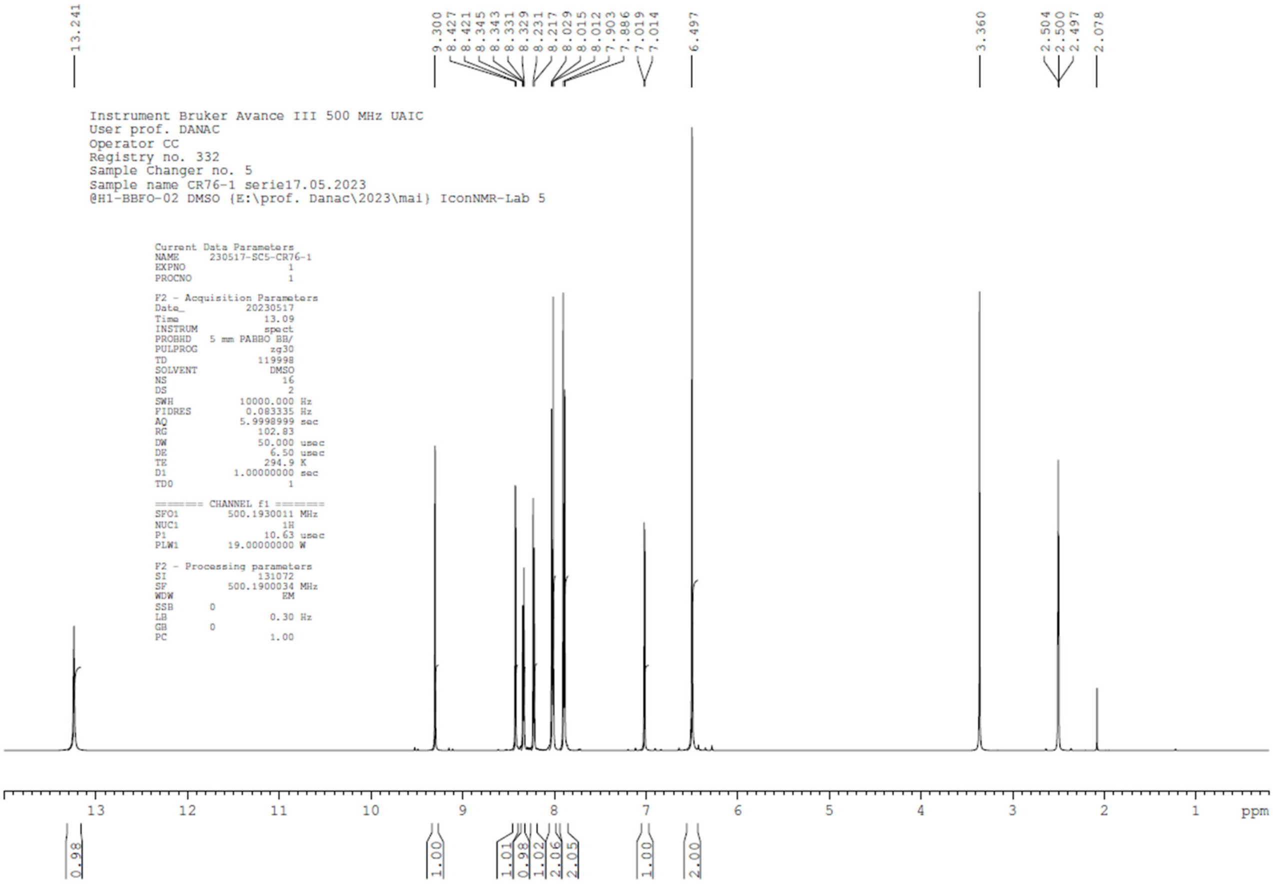

Figure S3. ^{1}H-NMR (500 MHz, DMSO-d$_6$) spectrum of compound **2b**

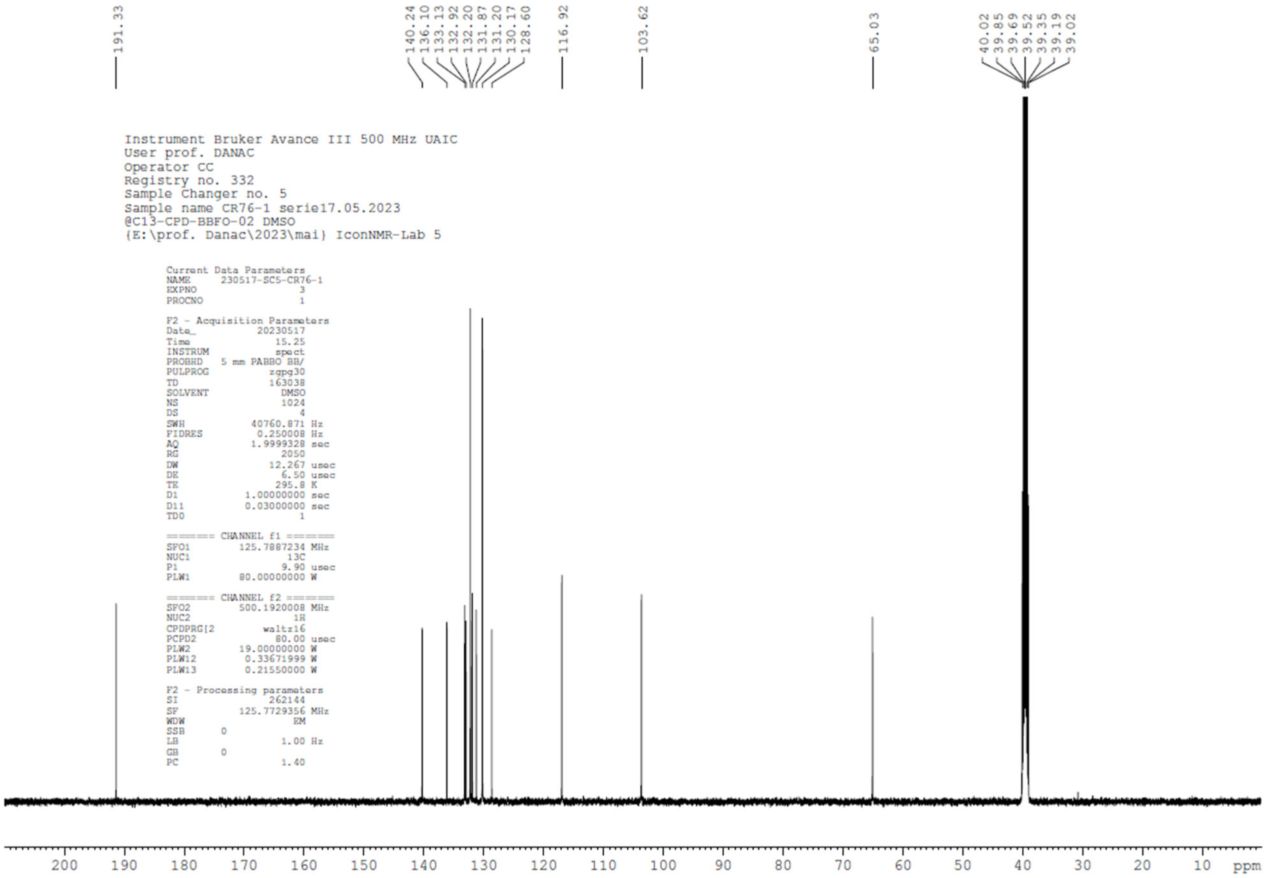

Figure S4. ^{13}C-NMR (125 MHz, DMSO-d$_6$) spectrum of compound **2b**

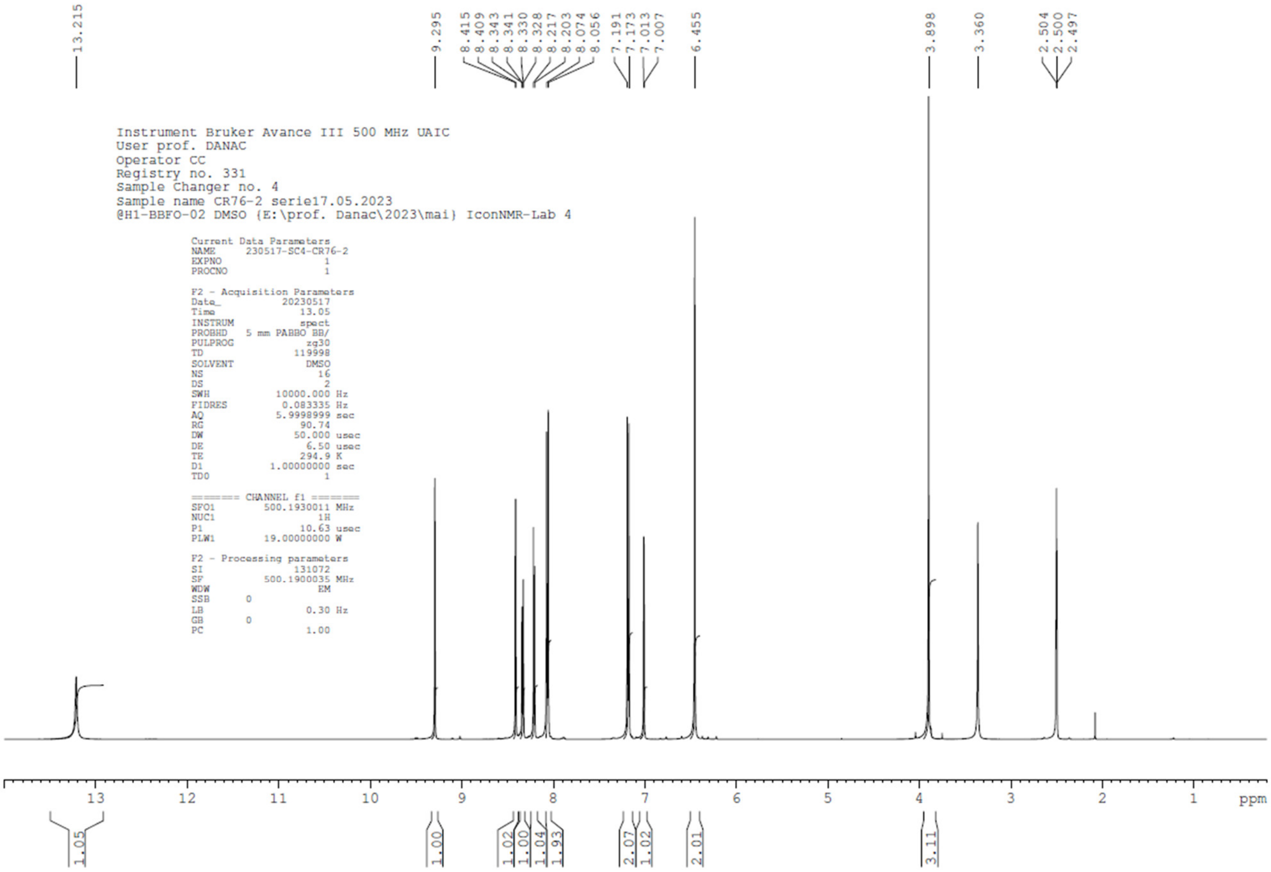

Figure S5. ^{1}H-NMR (500 MHz, DMSO-d$_6$) spectrum of compound **2c**

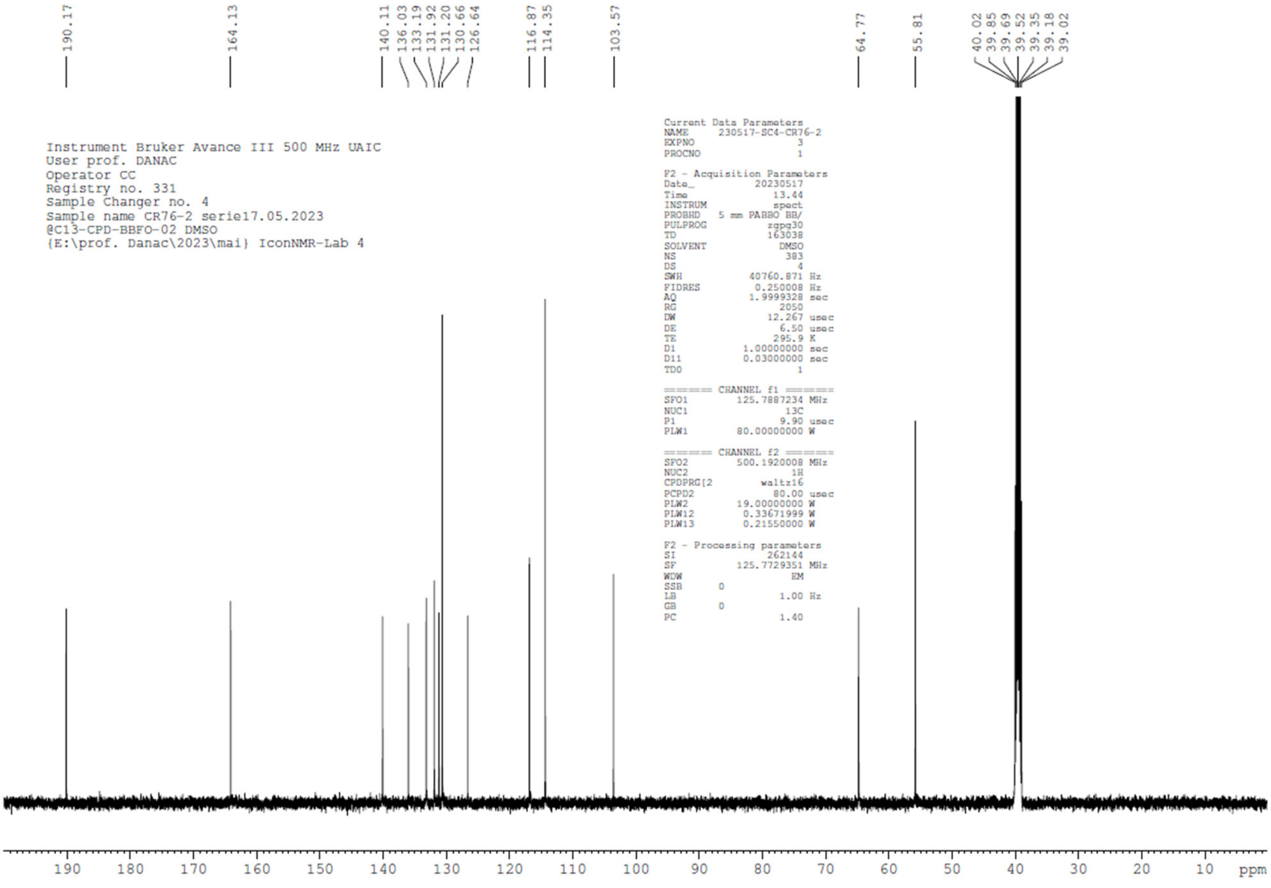

Figure S6. ^{13}C-NMR (125 MHz, DMSO-d$_6$) spectrum of compound **2c**

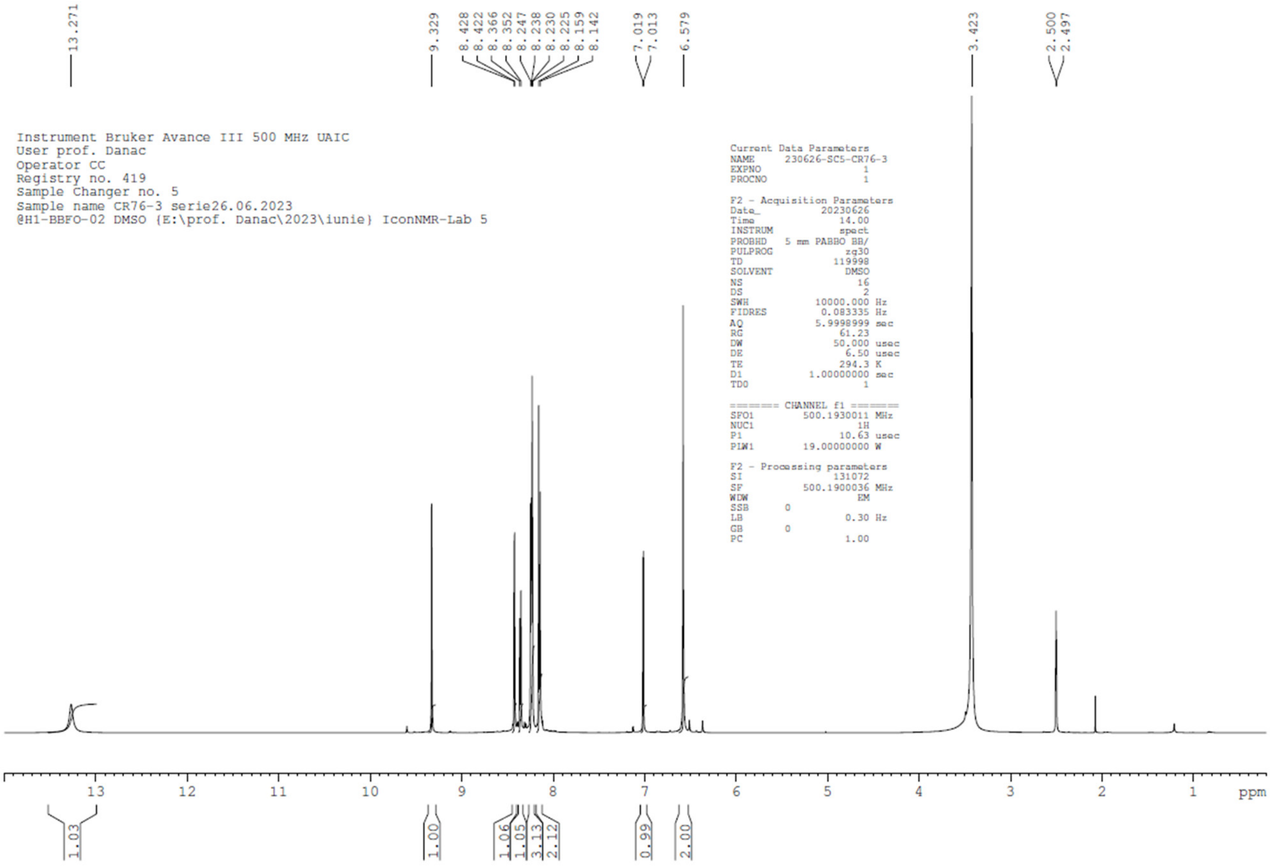

Figure S7. ^{1}H-NMR (500 MHz, DMSO-d$_6$) spectrum of compound **2d**

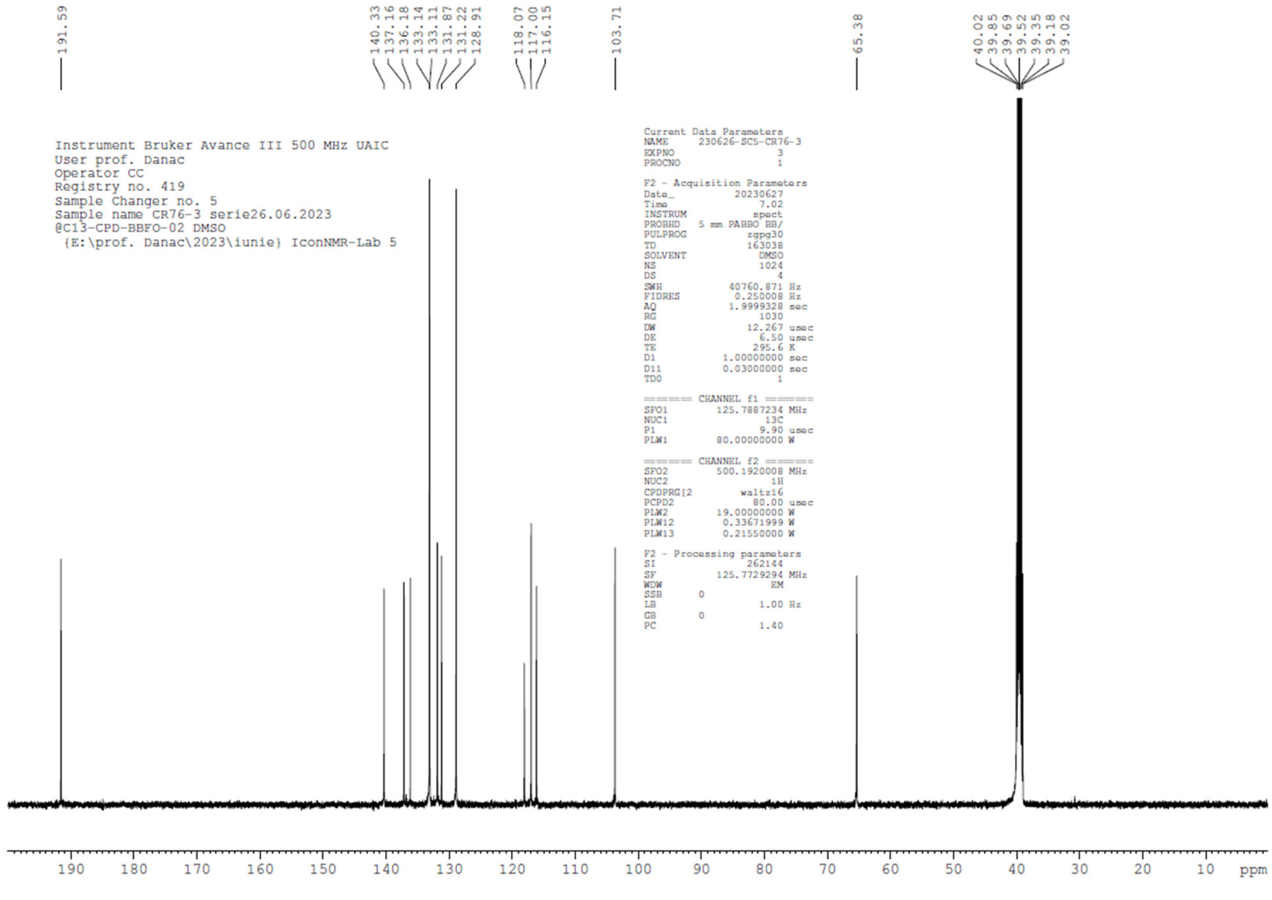

Figure S8. ^{13}C-NMR (125 MHz, DMSO-d$_6$) spectrum of compound **2d**

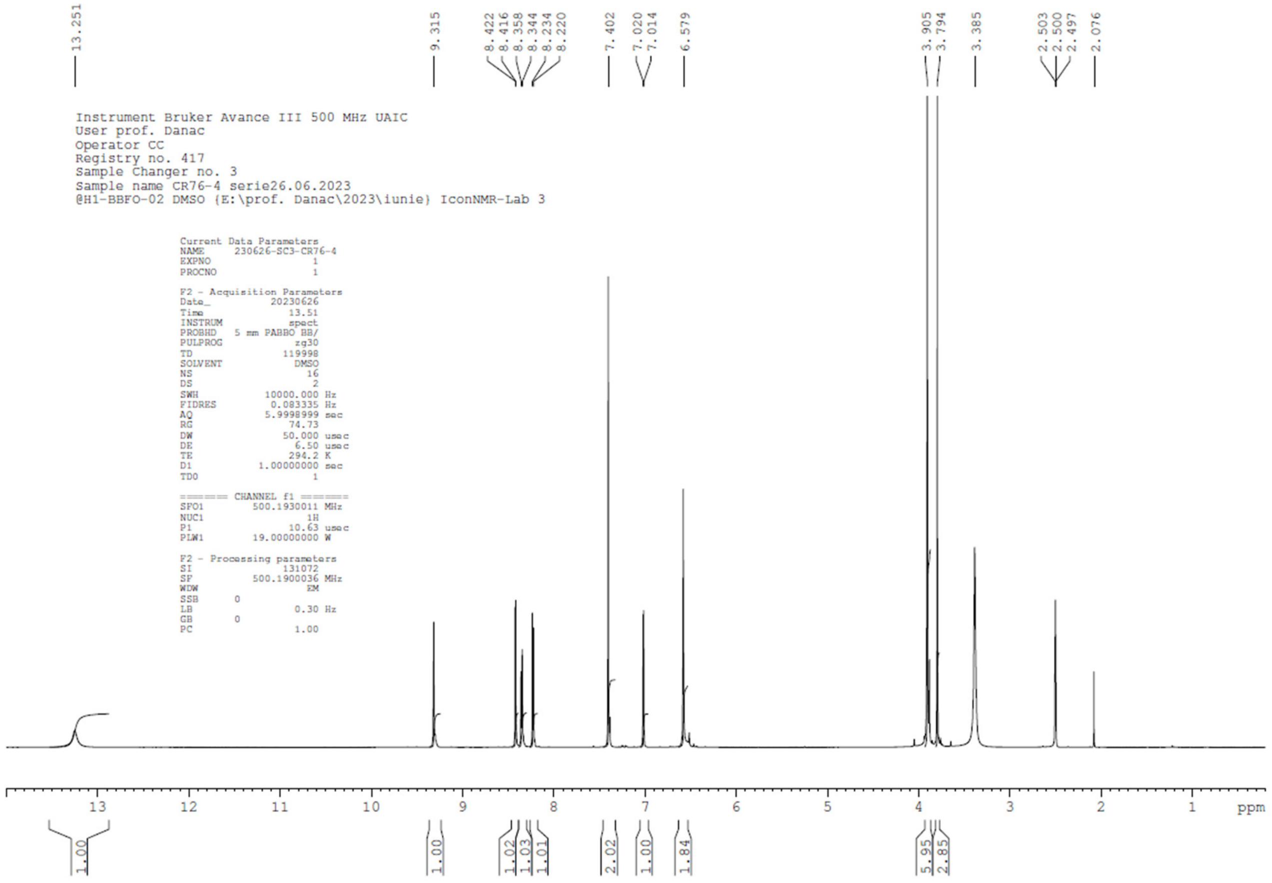

Figure S9. ^{1}H-NMR (500 MHz, DMSO-d6) spectrum of compound **2e**

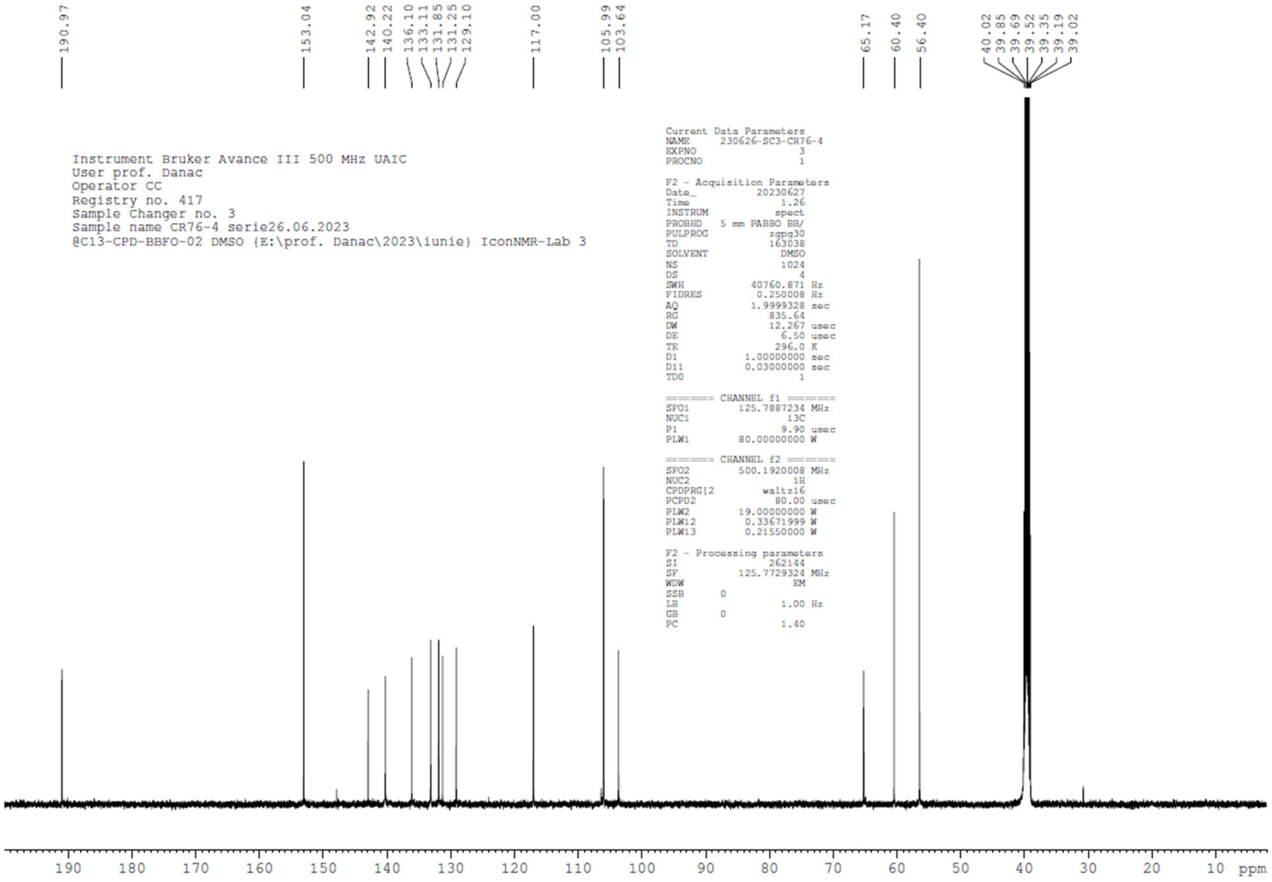

Figure S10. ^{13}C-NMR (125 MHz, DMSO-d6) spectrum of compound **2e**

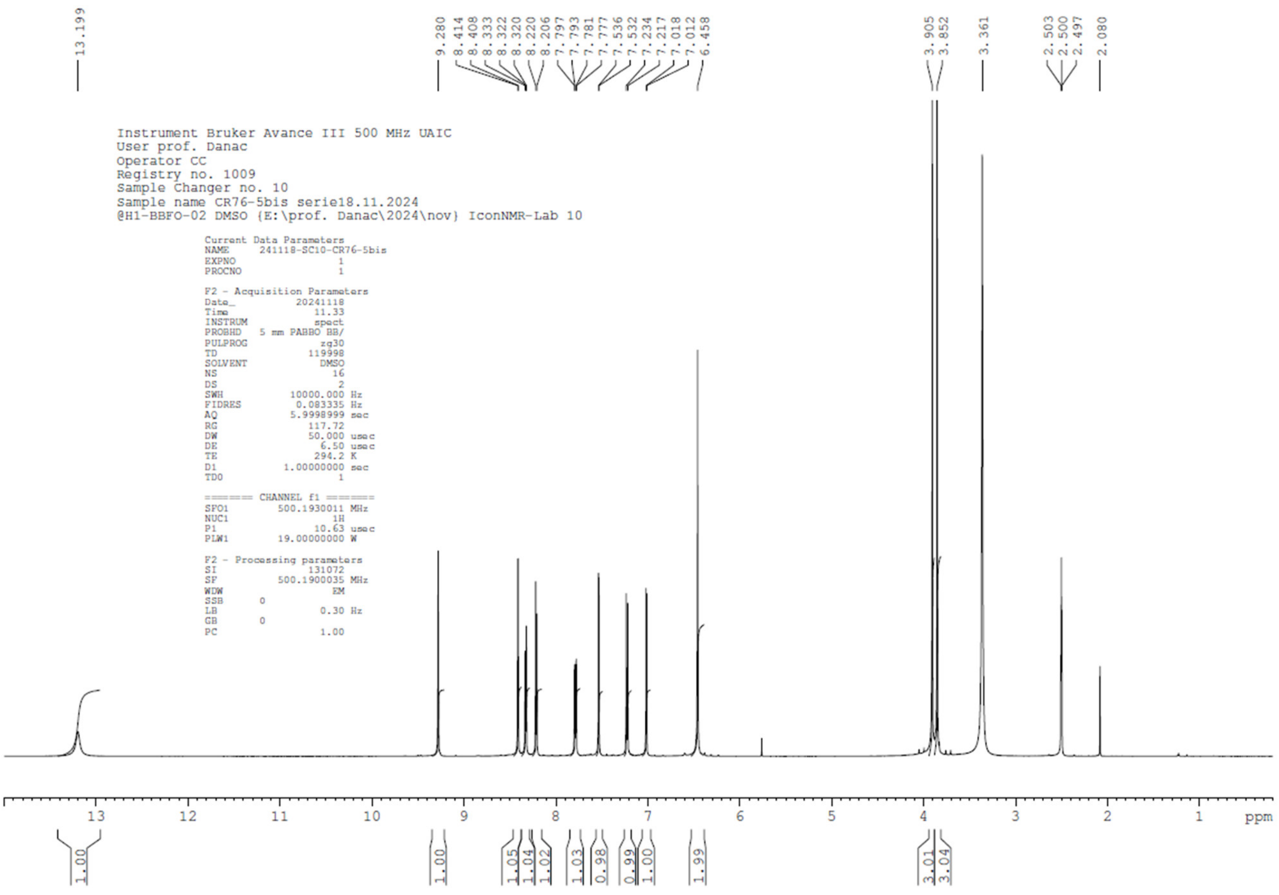

Figure S11. ^{1}H-NMR (500 MHz, DMSO-d6) spectrum of compound **2f**

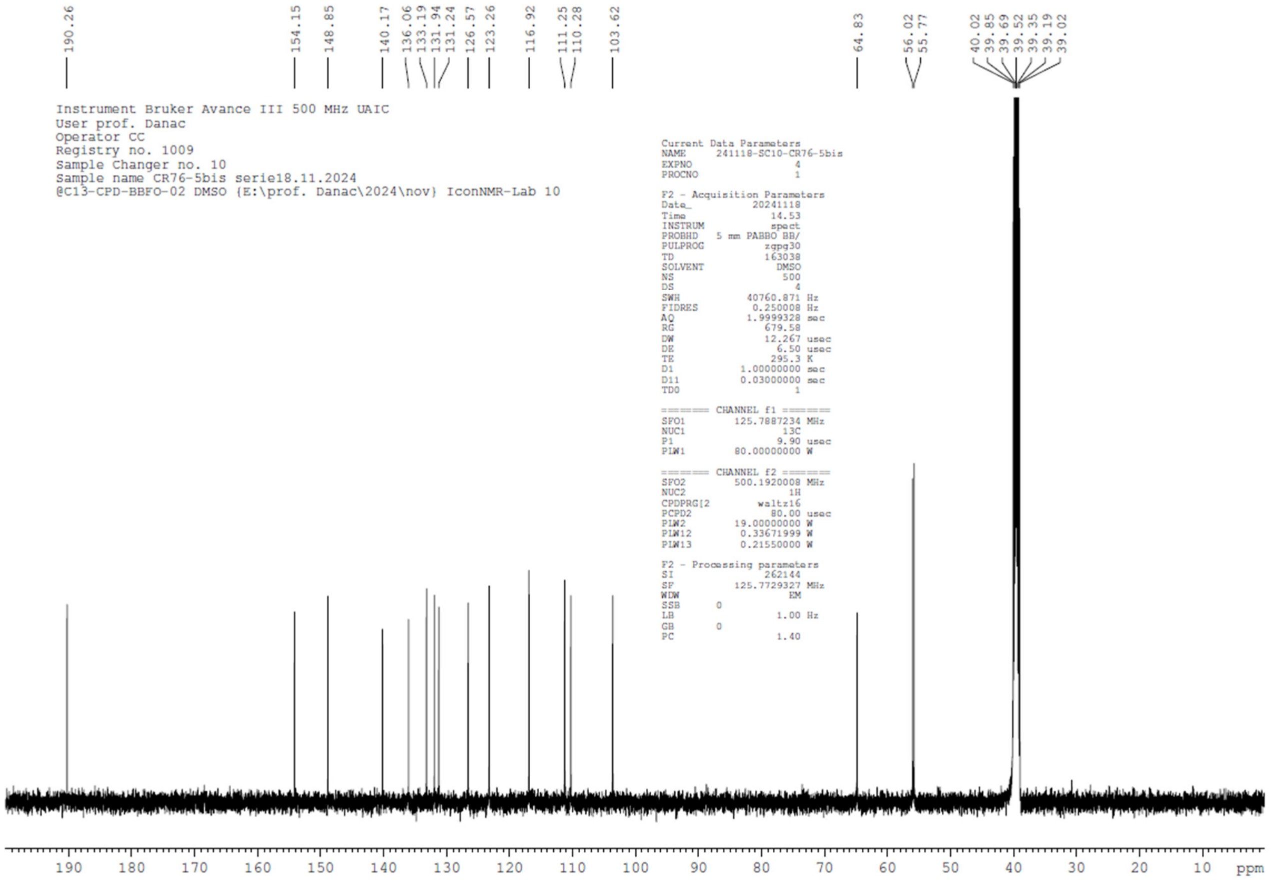

Figure S12. ^{13}C-NMR (125 MHz, DMSO-d6) spectrum of compound **2f**

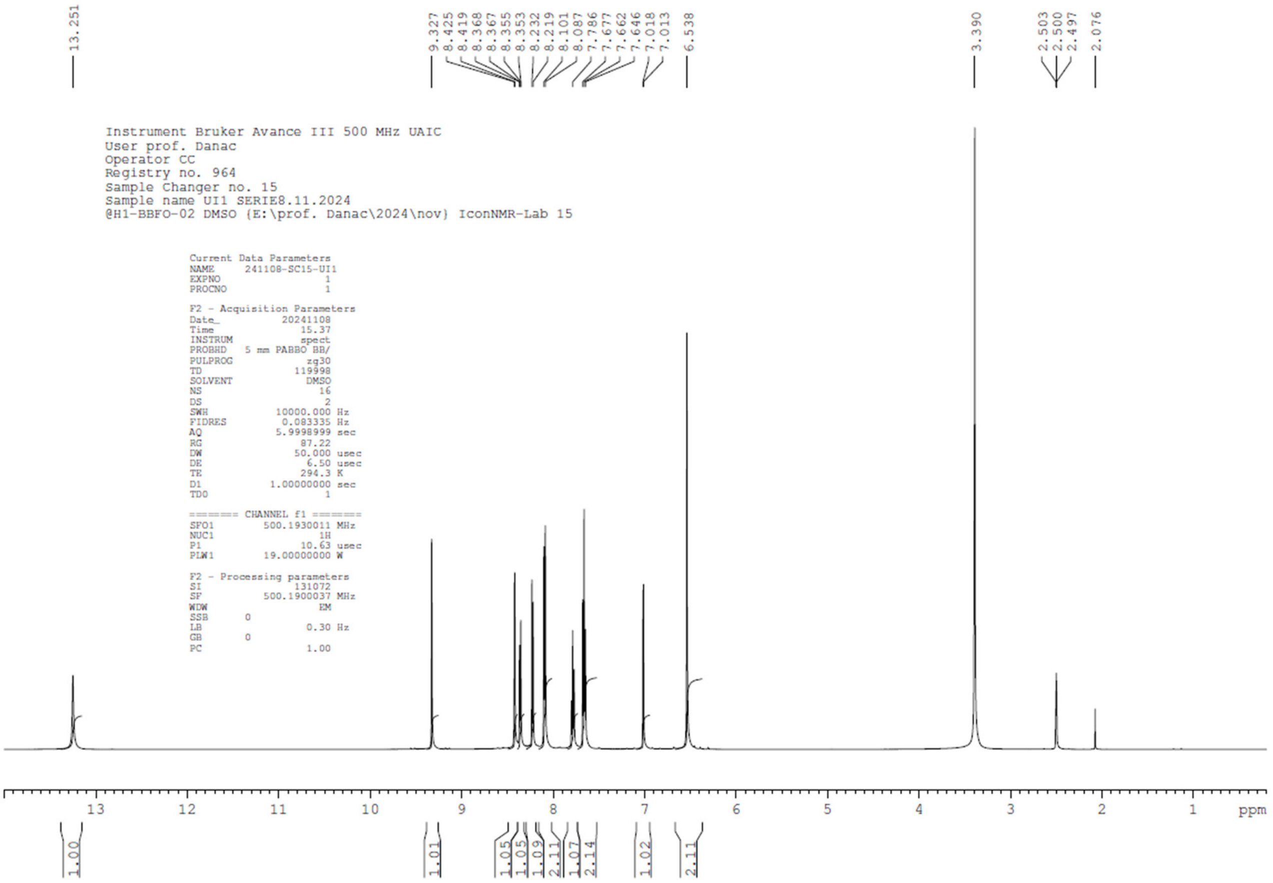

Figure S13. ^{1}H-NMR (500 MHz, DMSO-d$_6$) spectrum of compound **2g**

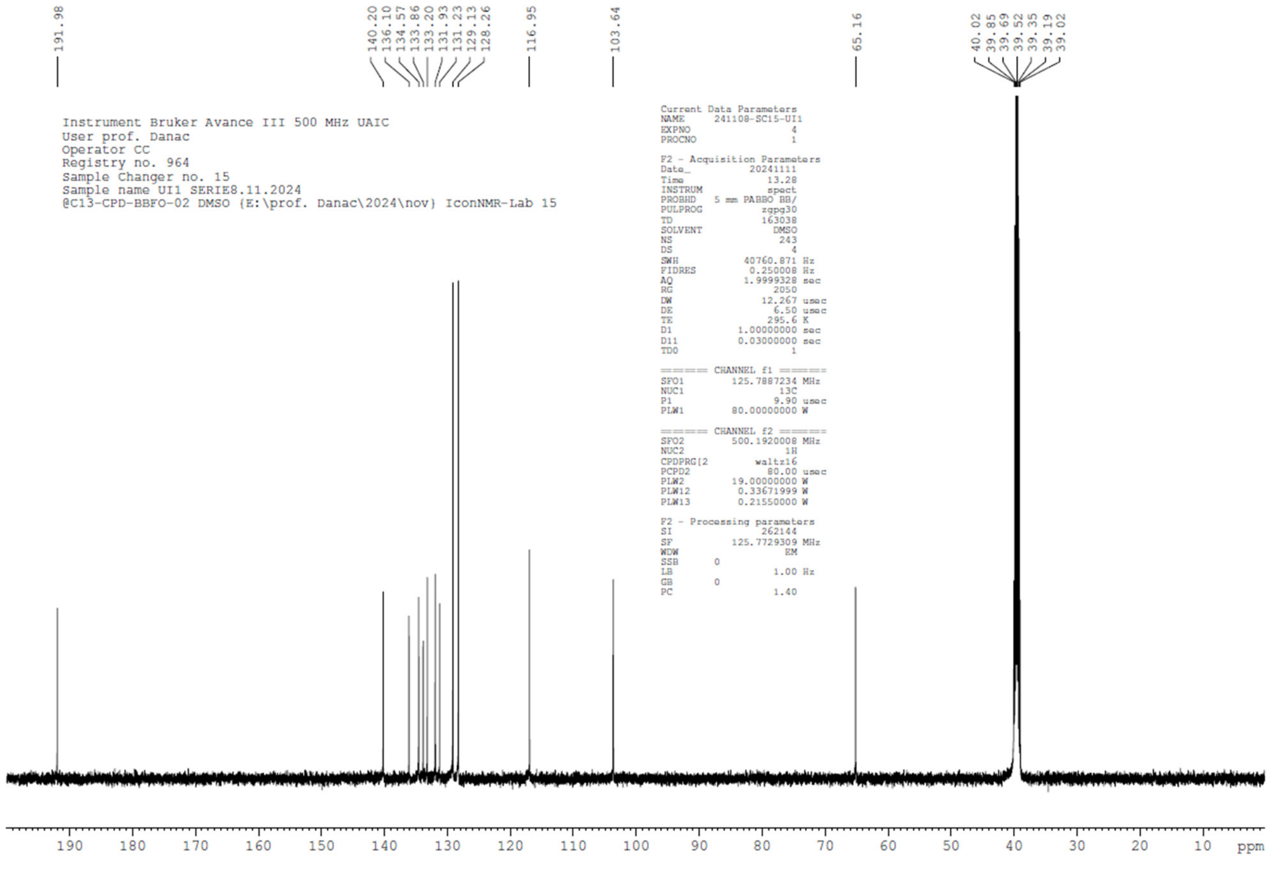

Figure S14. ^{13}C-NMR (125 MHz, DMSO-d$_6$) spectrum of compound **2g**

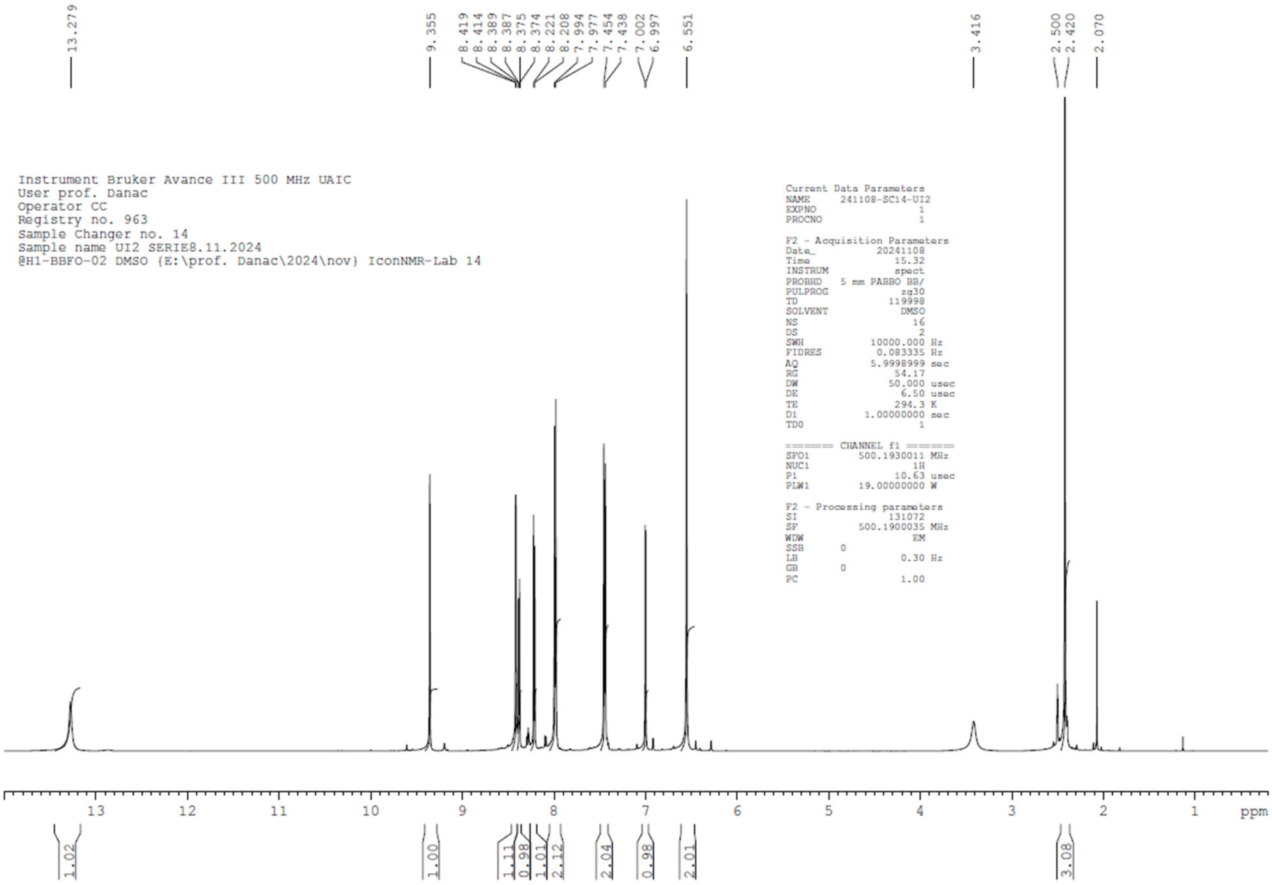

Figure S15. ^{1}H-NMR (500 MHz, DMSO-d$_6$) spectrum of compound **2h**

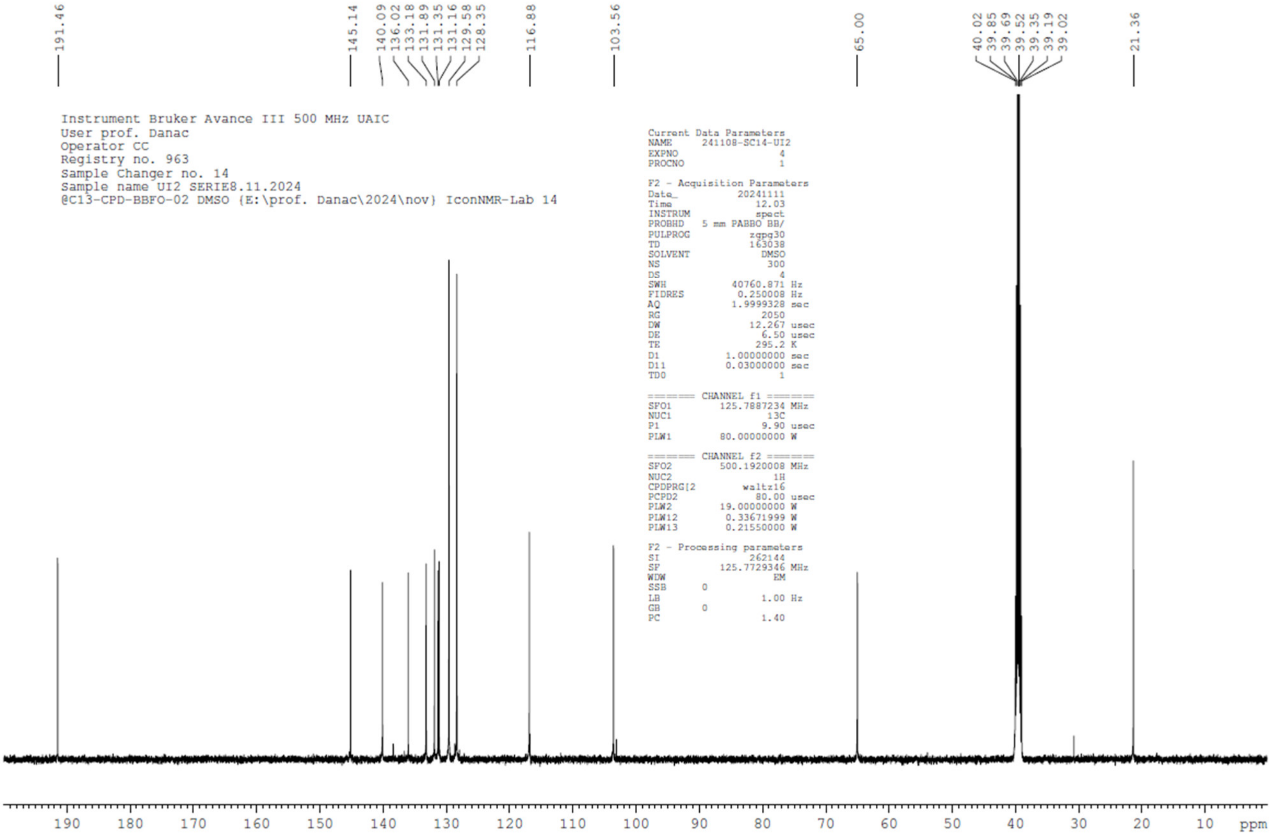

Figure S16. ^{13}C-NMR (125 MHz, DMSO-d$_6$) spectrum of compound **2h**

Developmental Therapeutics Program One Dose Bar Graph	NSC: D-850835 / 1	Conc: 1.000E-5 M	Test Date: Jan 8, 2024
	Experiment ID: 2401HT23		Report Date: Feb 22, 2024

Panel/Cell Line	Growth Percent
Leukemia	
CCRF-CEM	101.74
HL-60(TB)	96.25
K-562	117.81
MOLT-4	98.02
RPMI-8226	109.88
Non-Small Cell Lung Cancer	
A549/ATCC	93.09
EKVX	98.27
HOP-62	111.45
HOP-92	108.41
NCI-H226	97.30
NCI-H23	90.79
NCI-H322M	104.13
NCI-H460	102.30
NCI-H522	118.15
Colon Cancer	
COLO 205	113.72
HCC-2998	107.58
HCT-116	96.00
HCT-15	103.25
HT29	91.86
KM12	102.37
SW-620	95.07
CNS Cancer	
SF-268	106.20
SF-295	100.81
SF-539	101.83
SNB-19	104.20
SNB-75	104.63
U251	105.70
Melanoma	
LOX IMVI	81.64
MALME-3M	98.23
M14	106.77
MDA-MB-435	105.76
SK-MEL-2	102.25
SK-MEL-28	108.36
SK-MEL-5	91.79
UACC-257	112.25
UACC-62	108.52
Ovarian Cancer	
IGROV1	96.81
OVCAR-3	96.22
OVCAR-4	105.96
OVCAR-8	95.08
NCI/ADR-RES	108.81
SK-OV-3	100.83
Renal Cancer	
786-0	98.97
A498	102.25
ACHN	101.18
CAKI-1	102.84
RXF 393	93.07
SN12C	98.42
TK-10	105.62
Prostate Cancer	
PC-3	105.45
DU-145	106.15
Breast Cancer	
MCF7	102.93
HS 578T	99.04
BT-549	101.69
MDA-MB-468	76.76

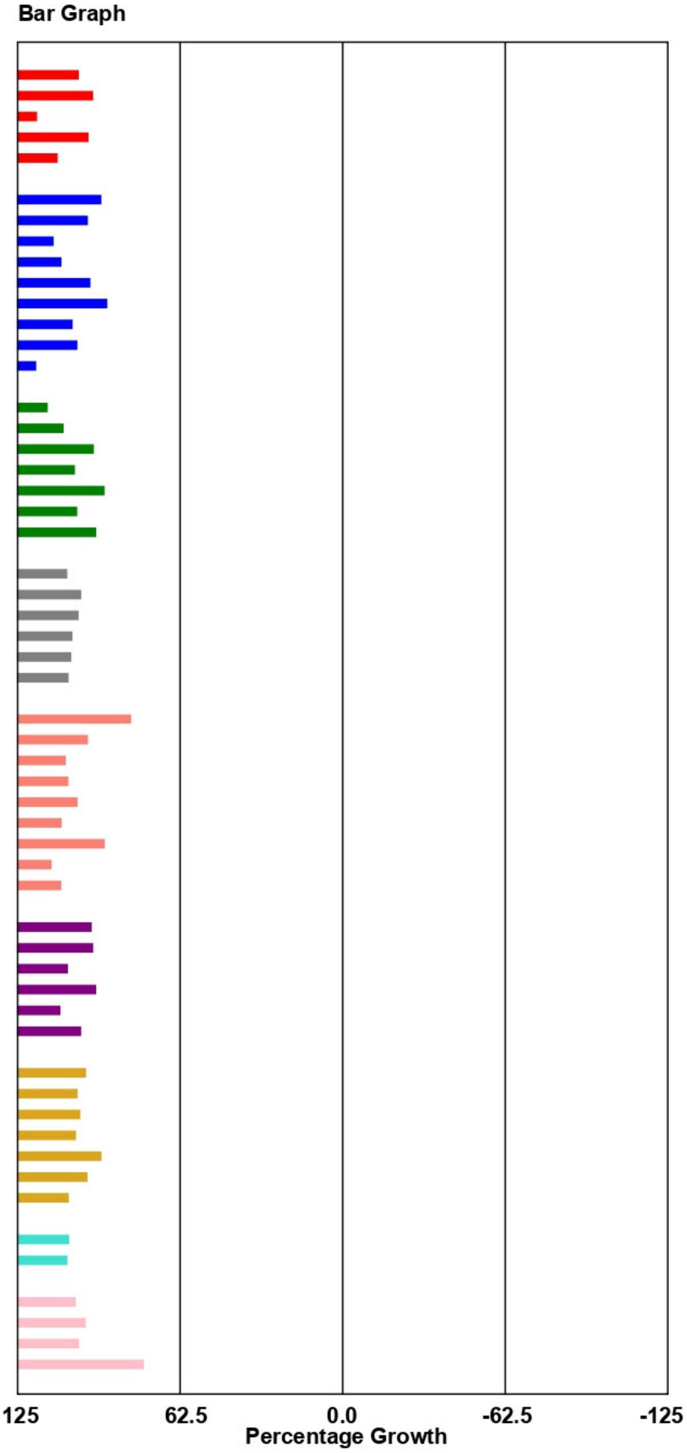

Figure S17. Results of the *in vitro* growth of cancer cell lines in the single-dose assay for compound **2a**

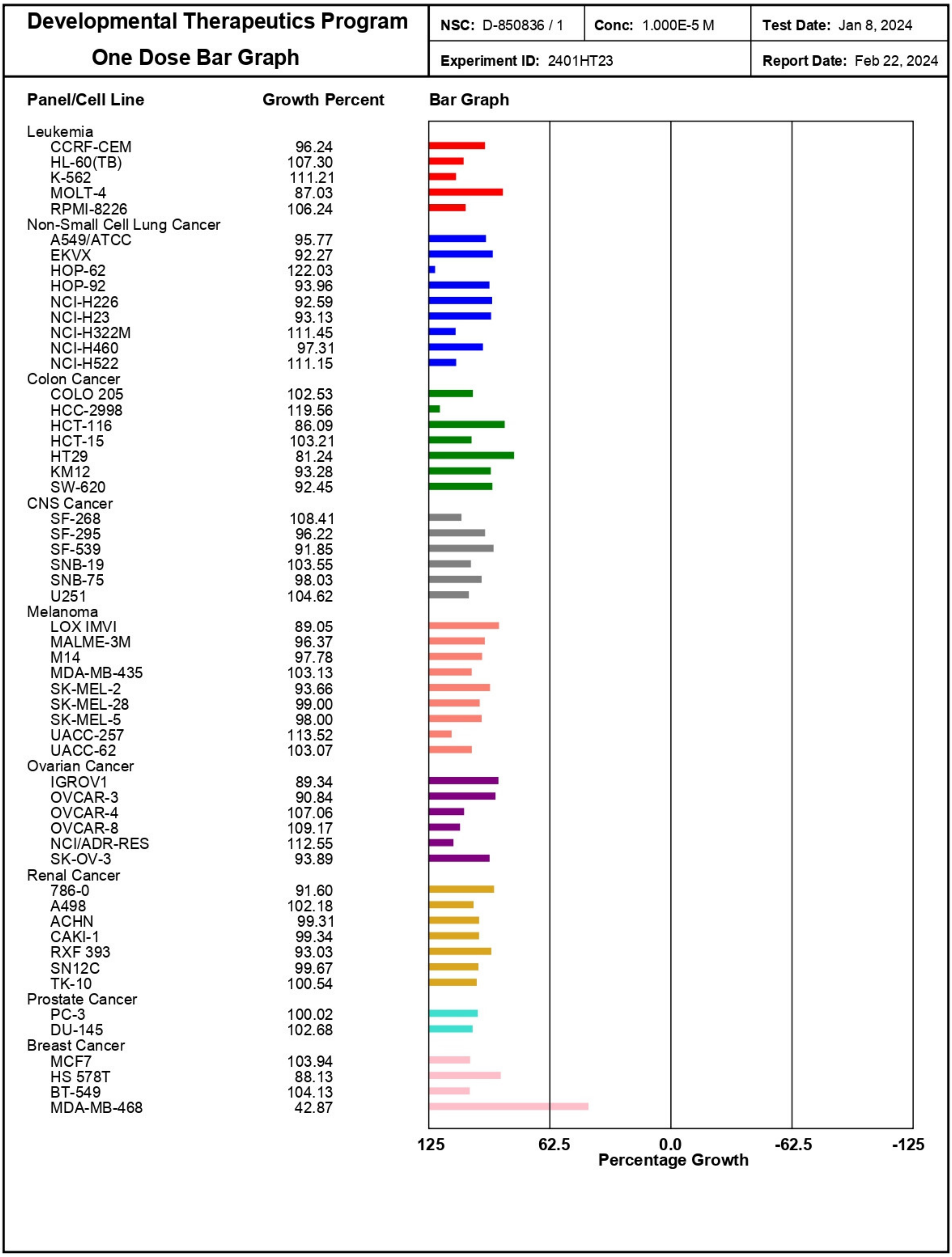

Developmental Therapeutics Program
One Dose Bar Graph

NSC: D-850836 / 1 | **Conc:** 1.000E-5 M | **Test Date:** Jan 8, 2024
Experiment ID: 2401HT23 | **Report Date:** Feb 22, 2024

Panel/Cell Line	Growth Percent
Leukemia	
CCRF-CEM	96.24
HL-60(TB)	107.30
K-562	111.21
MOLT-4	87.03
RPMI-8226	106.24
Non-Small Cell Lung Cancer	
A549/ATCC	95.77
EKVX	92.27
HOP-62	122.03
HOP-92	93.96
NCI-H226	92.59
NCI-H23	93.13
NCI-H322M	111.45
NCI-H460	97.31
NCI-H522	111.15
Colon Cancer	
COLO 205	102.53
HCC-2998	119.56
HCT-116	86.09
HCT-15	103.21
HT29	81.24
KM12	93.28
SW-620	92.45
CNS Cancer	
SF-268	108.41
SF-295	96.22
SF-539	91.85
SNB-19	103.55
SNB-75	98.03
U251	104.62
Melanoma	
LOX IMVI	89.05
MALME-3M	96.37
M14	97.78
MDA-MB-435	103.13
SK-MEL-2	93.66
SK-MEL-28	99.00
SK-MEL-5	98.00
UACC-257	113.52
UACC-62	103.07
Ovarian Cancer	
IGROV1	89.34
OVCAR-3	90.84
OVCAR-4	107.06
OVCAR-8	109.17
NCI/ADR-RES	112.55
SK-OV-3	93.89
Renal Cancer	
786-0	91.60
A498	102.18
ACHN	99.31
CAKI-1	99.34
RXF 393	93.03
SN12C	99.67
TK-10	100.54
Prostate Cancer	
PC-3	100.02
DU-145	102.68
Breast Cancer	
MCF7	103.94
HS 578T	88.13
BT-549	104.13
MDA-MB-468	42.87

Figure S18. Results of the *in vitro* growth of cancer cell lines in the single-dose assay for compound **2b**

Developmental Therapeutics Program

One Dose Bar Graph

**NSC:** D-850885 / 1	**Conc:** 1.000E-5 M
**Test Date:** Jan 8, 2024	**Experiment ID:** 2401HT23
**Report Date:** Feb 22, 2024	

Panel/Cell Line	Growth Percent
Leukemia	
CCRF-CEM	82.38
HL-60(TB)	84.77
K-562	122.06
MOLT-4	95.26
RPMI-8226	110.89
Non-Small Cell Lung Cancer	
A549/ATCC	98.73
EKVX	99.46
HOP-62	116.29
HOP-92	107.02
NCI-H226	92.45
NCI-H23	101.12
NCI-H322M	122.05
NCI-H460	102.76
NCI-H522	108.31
Colon Cancer	
COLO 205	118.06
HCC-2998	107.19
HCT-116	97.76
HCT-15	107.32
HT29	95.98
KM12	99.03
SW-620	103.41
CNS Cancer	
SF-268	108.35
SF-295	98.56
SF-539	95.93
SNB-19	108.09
SNB-75	87.46
U251	113.56
Melanoma	
LOX IMVI	87.12
MALME-3M	108.82
M14	104.74
MDA-MB-435	119.36
SK-MEL-2	99.83
SK-MEL-28	102.46
SK-MEL-5	98.25
UACC-257	117.55
UACC-62	116.21
Ovarian Cancer	
IGROV1	105.05
OVCAR-3	101.85
OVCAR-4	112.79
OVCAR-8	95.23
NCI/ADR-RES	110.51
SK-OV-3	102.69
Renal Cancer	
786-0	102.30
A498	99.78
ACHN	102.07
CAKI-1	104.85
RXF 393	91.53
SN12C	94.44
TK-10	107.00
Prostate Cancer	
PC-3	105.43
DU-145	105.95
Breast Cancer	
MCF7	99.49
HS 578T	96.71
BT-549	96.19
MDA-MB-468	110.84

Bar Graph

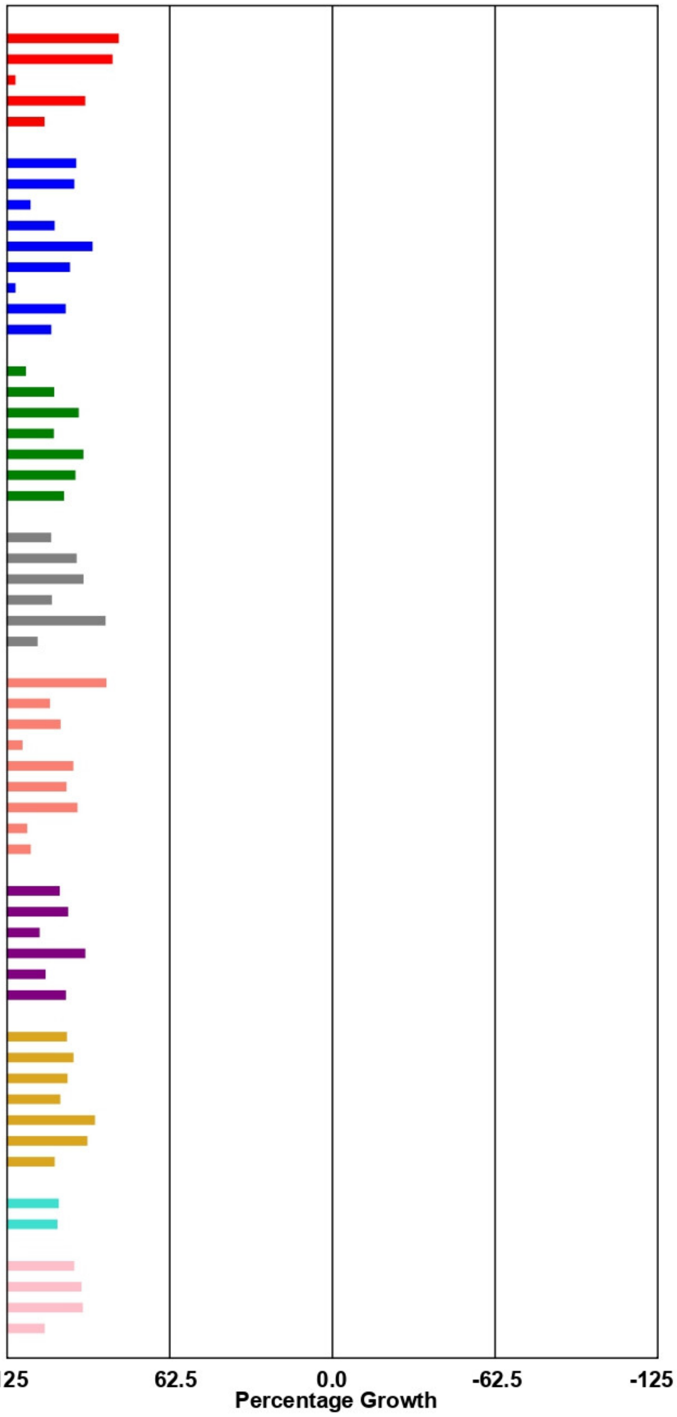

Figure S19. Results of the *in vitro* growth of cancer cell lines in the single-dose assay for compound **2c**

Developmental Therapeutics Program One Dose Bar Graph	NSC: D-850886 / 1	Conc: 1.000E-5 M	Test Date: Jan 8, 2024
	Experiment ID: 2401HT23		Report Date: Feb 22, 2024

Panel/Cell Line	Growth Percent
**Leukemia**	
CCRF-CEM	82.76
HL-60(TB)	96.34
K-562	125.15
MOLT-4	92.95
RPMI-8226	111.93
**Non-Small Cell Lung Cancer**	
A549/ATCC	103.24
EKVX	94.35
HOP-62	85.27
HOP-92	117.30
NCI-H226	108.62
NCI-H23	95.36
NCI-H322M	115.57
NCI-H460	102.26
NCI-H522	114.48
**Colon Cancer**	
COLO 205	120.90
HCC-2998	98.07
HCT-116	109.19
HCT-15	100.54
HT29	100.96
KM12	97.56
SW-620	107.37
**CNS Cancer**	
SF-268	109.05
SF-295	107.00
SF-539	96.61
SNB-19	108.86
SNB-75	95.89
U251	114.57
**Melanoma**	
LOX IMVI	91.35
MALME-3M	102.68
M14	106.62
MDA-MB-435	113.05
SK-MEL-2	97.78
SK-MEL-28	112.08
SK-MEL-5	99.15
UACC-257	123.40
UACC-62	115.95
**Ovarian Cancer**	
IGROV1	105.62
OVCAR-3	101.09
OVCAR-4	121.31
OVCAR-8	109.25
NCI/ADR-RES	119.39
SK-OV-3	112.07
**Renal Cancer**	
786-0	102.92
A498	109.89
ACHN	104.49
CAKI-1	108.12
RXF 393	99.77
SN12C	103.20
TK-10	102.69
**Prostate Cancer**	
PC-3	109.59
DU-145	106.75
**Breast Cancer**	
MCF7	97.65
HS 578T	94.17
BT-549	100.29
MDA-MB-468	113.87

Bar Graph

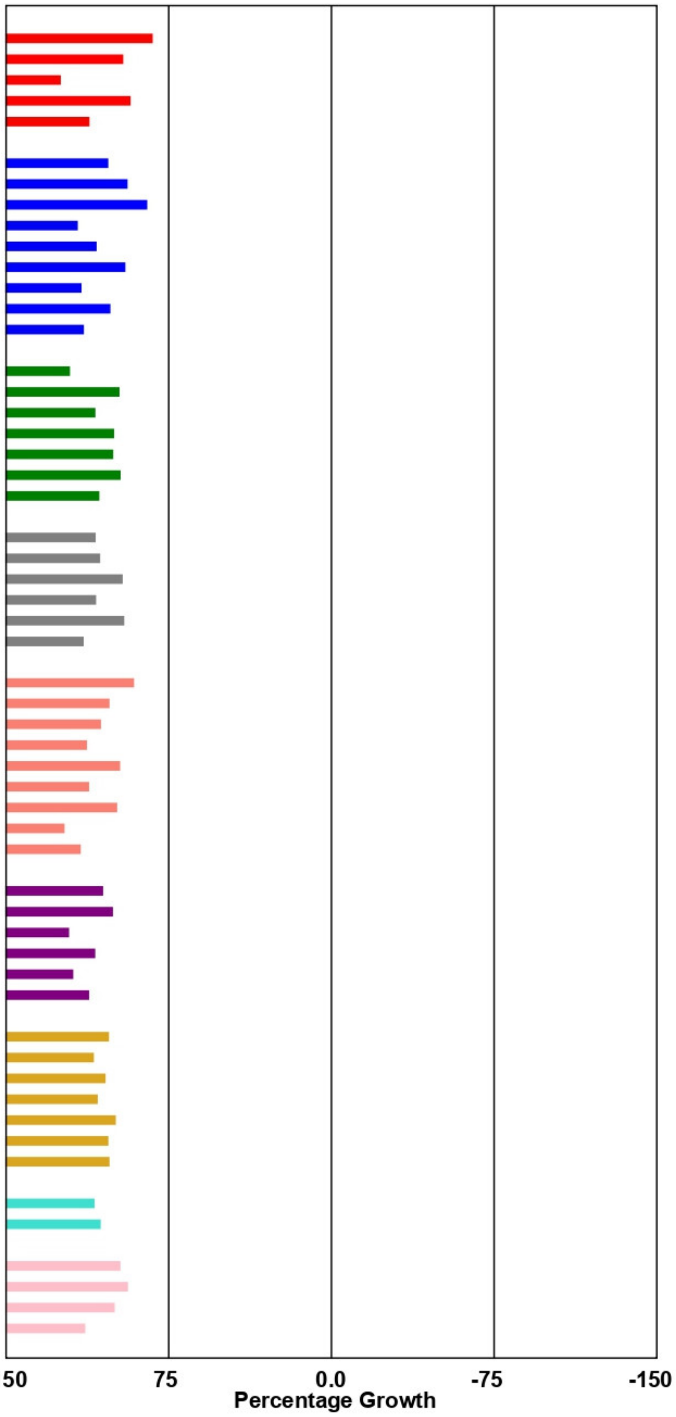

Figure S20. Results of the *in vitro* growth of cancer cell lines in the single-dose assay for compound **2d**

Developmental Therapeutics Program One Dose Bar Graph	NSC: D-850887 / 1	Conc: 1.000E-5 M	Test Date: Jan 8, 2024
	Experiment ID: 2401HT23		Report Date: Feb 22, 2024

Panel/Cell Line	Growth Percent
**Leukemia**	
CCRF-CEM	88.36
HL-60(TB)	87.07
K-562	129.18
MOLT-4	97.48
RPMI-8226	119.24
**Non-Small Cell Lung Cancer**	
A549/ATCC	99.28
EKVX	99.86
HOP-62	104.27
HOP-92	114.28
NCI-H226	108.40
NCI-H23	99.55
NCI-H322M	113.78
NCI-H460	104.25
NCI-H522	103.00
**Colon Cancer**	
COLO 205	117.83
HCC-2998	91.29
HCT-116	106.07
HCT-15	102.82
HT29	103.35
KM12	106.45
SW-620	106.34
**CNS Cancer**	
SF-268	107.94
SF-295	101.13
SF-539	98.10
SNB-19	110.21
SNB-75	96.73
U251	112.61
**Melanoma**	
LOX IMVI	87.05
MALME-3M	113.76
M14	101.23
MDA-MB-435	117.80
SK-MEL-2	91.79
SK-MEL-28	103.90
SK-MEL-5	100.00
UACC-257	117.26
UACC-62	108.38
**Ovarian Cancer**	
IGROV1	101.11
OVCAR-3	98.95
OVCAR-4	122.60
OVCAR-8	104.99
NCI/ADR-RES	117.44
SK-OV-3	108.07
**Renal Cancer**	
786-0	101.12
A498	107.42
ACHN	98.38
CAKI-1	101.37
RXF 393	92.23
SN12C	94.64
TK-10	94.17
**Prostate Cancer**	
PC-3	110.04
DU-145	104.65
**Breast Cancer**	
MCF7	109.92
HS 578T	92.83
BT-549	100.99
MDA-MB-468	146.81

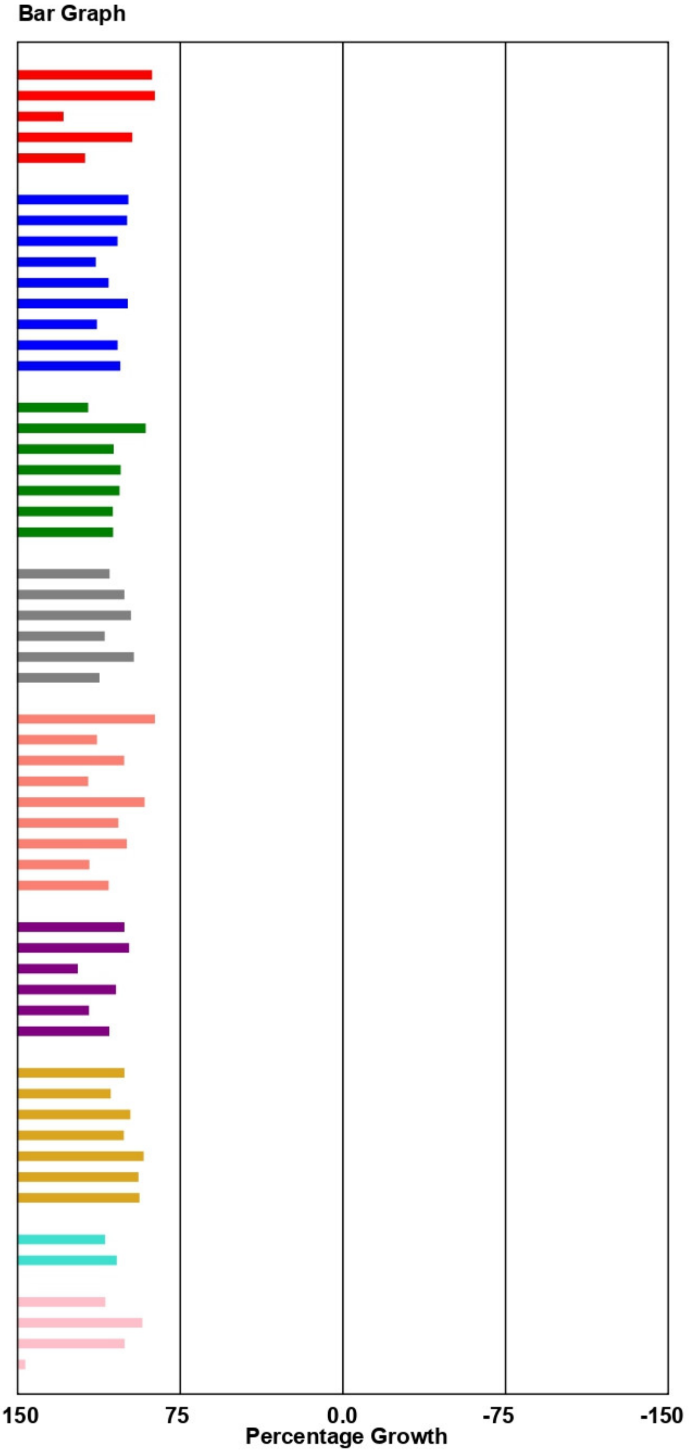

Figure S21. Results of the *in vitro* growth of cancer cell lines in the single-dose assay for compound **2e**
